# Recapitulation of prostate tissue cell type-specific transcriptomes by an *in vivo* primary prostate tissue xenograft model

**DOI:** 10.1371/journal.pone.0233899

**Published:** 2020-06-25

**Authors:** Nelson T. Gross, Jianmin Wang, Michael V. Fiandalo, Eduardo Cortes Gomez, Anica Watts, Alejandro S. Godoy, Gary J. Smith, Yue Wu

**Affiliations:** 1 Department of Urology, Roswell Park Comprehensive Cancer Center, Buffalo, New York, United States of America; 2 Department of Biostatistics and Bioinformatics, Roswell Park Comprehensive Cancer Center, Buffalo, New York, United States of America; 3 Department of Physiology, Pontificial Universidad Católica de Chile, Santiago, Chile; 4 Centro de Biología Celular y Biomedicina (CEBICEM), Universidad San Sebastián, Santiago, Chile; Universidad de Jaen, SPAIN

## Abstract

Studies of the normal functions and diseases of the prostate request *in vivo* models that maintain the tissue architecture and the multiple-cell type compartments of human origin in order to recapitulate reliably the interactions of different cell types. Cell type-specific transcriptomes are critical to reveal the roles of each cell type in the functions and diseases of the prostate. A primary prostate tissue xenograft model was developed using fresh human prostate tissue specimens transplanted onto male mice that were castrated surgically and implanted with a device to maintain circulating testosterone levels comparable to adult human males. Endothelial cells and epithelial cells were isolated from 7 fresh human prostate tissue specimens and from primary tissue xenografts established from 9 fresh human prostate tissue specimens, using antibody-conjugated magnetic beads specific to human CD31 and human EpCAM, respectively. Transcriptomes of endothelial, epithelial and stromal cell fractions were obtained using RNA-Seq. Global and function-specific gene expression profiles were compared in inter-cell type and inter-tissue type manners. Gene expression profiles in the individual cell types isolated from xenografts were similar to those of cells isolated from fresh tissue, demonstrating the value of the primary tissue xenograft model for studies of the inter-relationships between prostatic cell types and the role of such inter-relationships in organ development, disease progression, and response to drug treatments.

## Introduction

Organogenesis and post-pubertal development of the prostate, as well as maintenance of tissue architecture and function in the adult prostate, are highly coordinated processes that involve interactions of multiple cell-type compartments, including endothelial, stromal and epithelial cells, that are tightly regulated by androgen [1, 2]. Importantly, these complex interactions among different cells types also play critical roles in development and progression of prostate cancer [3–6]. Prostate cancer cell lines and organoids of human origin, and their respective *in vivo* xenografts, have proven to be valuable models for the study of signaling and metabolic pathways in cancer epithelial cells [7–10]. However, the lack of other human cell-compartments and tissue architecture limits the use of these models for investigation of the regulatory role of the tumor microenvironment, and the role of cell-cell and cell-microenvironment interactions, in development and maintenance of the adult organ and in disease progression. Co-culture systems comprised of mixtures of multiple prostate cell types are a step forward compared to cell lines and organoids, but still lack the tissue architecture and the complex cell-cell interactions that exist in human prostate tissue [11]. Short-term cultures of fresh tissue preserve the cellular diversity and tissue architecture of benign or malignant human prostate tissue, therefore, studies using fresh tissue *ex vivo* could add significant new knowledge of the prostate tissue microenvironment [12]. However, *ex vivo* cultures are short-lived and all cells of the small pieces of tissue are bathed equally in substrates, obviating the role of vascular selectivity on regulated access of substrates or metabolites to internal cells, on juxtacrine/paracrine signaling and on differential metabolic capabilities of the different cell types in the tissue *in vivo*. Patient tissue-derived, serially passaged xenografts (PDX) of human tissue/cancers are valued *in vivo* models for the study of the response of the epithelial compartment to stimuli or therapeutic agents [13–16]. However, serially passaged PDX models lack the human endothelial cells of the human tissue micro-vasculature that provide a critical barrier between circulating signaling molecules/hormones/drugs and the prostate tissue microenvironment and epithelial cells. Furthermore, prostate endothelial cells and epithelial cells are inter-related functionally via critical endocrine and paracrine pathways, and prostate endothelial cells potentially are an essential component of the stem cell niche [17–19]. Similarly, a lack of human vasculature also hampers studies of human endothelial cell-cancer cell interactions in transgenic mouse tumor models.

Primary tissue xenografts of intact fresh clinical tissue specimens provide the most valuable model for characterization of the role of the human prostate endothelial and stromal compartments in the regulation of availability and regulatory effects of systemically available signals/hormones/drugs, and the modulatory role of the endothelial and stromal compartments in tissue homeostasis [20]. Further, primary tissue xenografts provide a unique model for studying sequential time points after manipulation within specimens of a single patient, or comparison of inter-patient variability without the time and expense needed to establish and characterize multiple independent primary cell lines/organoids.

Cell type-specific gene expression profile analysis represents a powerful tool to validate the conservation of an *in vivo* physiologic state in primary tissue xenografts after maintenance on a mouse host. However, there are few reports of gene expression profiles in fresh prostate epithelial cells against which to benchmark the expression profiles of cells isolated from primary xenografts [21–23], and gene expression profiles of isolated endothelial cells and stromal cells from fresh prostate tissue currently are not available. In published studies that characterized prostate epithelial cell gene expression profiles, epithelial cells were isolated using antibodies specific for the epithelial cell markers Trop or EpCAM, and fluorescence-activated cell sorting [21, 22], or laser capture micro-dissection [23], and the gene expression profiles were obtained using RNA-Seq, qRT-PCR array or mRNA array.

The present study was designed to validate the utility of short-term primary tissue xenografts (maintained for 30 days on the humanized mouse host) as a model of the complex microenvironment of intact human prostate tissue, particularly the role of micro-vascular endothelial cells. A primary prostate tissue xenograft model was developed that used fresh clinical human prostate tissue specimens transplanted onto male mice that were surgically castrated and implanted with a device to continuously-release testosterone (T) to maintain circulating T levels comparable to adult human males [20]. Endothelial cells and epithelial cells were isolated from clinical specimens of fresh human prostate tissue, and from primary tissue xenografts established from intact fresh human prostate tissue, using human EpCAM-specific antibody-conjugated magnetic beads, and human CD31-specific antibody-conjugated magnetic beads, to recover human epithelial cells and human endothelial cells, respectively. The remaining cell population after the two rounds of immuno-bead selection was termed “stromal cells”. Transcriptomes of endothelial, epithelial and stromal cell fractions from both fresh tissue and tissue xenografts were obtained using RNA-Seq. Global and function-specific gene expression profiles were compared between the three cell types in fresh tissue specimens and in tissue xenografts, and between the same individual cell type isolated from fresh tissue specimens versus from tissue xenograft specimens. Gene expression profiles in the individual cell types isolated from xenografts maintained for 30 days *in vivo* were very similar to those of cells isolated from fresh tissue, suggesting the value of the primary tissue xenograft model for studies of the inter-relationship between prostatic cell types and the role of such inter-relationship in organ development, drug response, and disease progression, particularly of androgen deprivation therapy (ADT) for treatment of prostate cancer.

## Materials and methods

### Procurement of surgical remnant prostate tissue

Fresh benign prostate tissue specimens were procured from the Pathology Network Shared Resources (PNSR) at Roswell Park Comprehensive Cancer Center (RPCCC). The protocol (MOD00001531 / NHR 005009) for the use of fresh prostate tissue specimens was approved by the Institutional Review Board (IRB). The data were analyzed anonymously so consent was not obtained.

### Primary prostate tissue xenograft

Male Hsd:Athymic Nude-Fox1nu nude mice (12 weeks of age) were purchased from Envigo (Indianapolis, IN) [24]. Mice were castrated surgically and implanted subcutaneously with silastic tubing (catalog number 508–008 from VWR, Radnor, PA) manually packed with dry testosterone (T) crystals 3–5 days before transplantation with fresh tissue. This procedure standardized the levels of circulating T between mice at levels comparable to circulating T in adult male humans, (i.e., “humanized” mouse hosts) [25]. All experimental procedures were approved by the RPCCC Institutional Animal Care and Use Committee (IACUC) and were performed by trained staff of the RPCCC Mouse Tumor Model Resource (MTMR) core facility under IACUC protocol 1044M. Animals were anesthetized using Isoflurane via an anesthesia machine (provided by LAR) in the plastic chamber then transferred to the nose cone. Animals euthanized by deep plane anesthesia followed by exsanguination. Fresh prostate tissue specimens were received from the RPCCC PNSR in transport medium that contained Cellgro phenol red-free RPMI1640 medium from Corning life Sciences (Corning, NY) supplemented with 10% charcoal-stripped fetal bovine serum (CS-FBS); CS-FBS was prepared as described [26]. Tissue was cut into cubes 2–3 mm per side, pieces of tissue to be transplanted were coated with Matrigel from Corning Life Sciences, and the pieces were inserted individually at the subcutaneous site. A total of 5–8 pieces of tissue were transplanted to each “humanized” mouse. The resulting xenografts were considered to be established stably after 30 days [20, 27]. As demonstrated in previous study, the prostate tissue maintained human prostate tissue architecture and, most importantly, the glandular architecture, throughout the 14 day time course after T-deprivation [24]. At harvest, the host was anesthetized, the vascular contents were replaced by perfusion with sterile saline, and the tissue xenografts were removed surgically and placed in cold transport medium.

### Tissue disaggregation

The protocol for enzymatic disaggregation of prostate tissue to yield single cells was described previously [20]. Fresh prostate tissue specimens (1–4 g), or freshly harvested tissue xenograft specimens (pool of ~40 xenografts from 5–8 mouse hosts), were rinsed with PBS, and minced in phenol red-free RPMI medium supplemented with 10% CS-FBS using razor blades. Minced tissue was disaggregated to single cells using a combination of proteases: 1.0 mg/mL Collagenase Type 4 from Worthington Biochemical (Lakewood, NJ) and 0.22 mg/mL Neutral protease from Worthington Biochemical, and incubated at 37°C for 60 minutes. Protease digestion was stopped by low-speed centrifugation of the disaggregation solution contents to pellet the cells and allow removal of the disaggregation buffer supernatant. The pellet was rinsed with HBSS and suspended in 10 mL of DNase buffer (5mM MgCl_2_, 5mM CaCl_2_, 0.5% BSA) containing 10 mg of DNase from Millipore Sigma (Burlington, MA). After the DNase incubation, the cell suspension was passed sequentially through nylon mesh screens with pores of Falcon 100 μm from Thermo Fisher Scientific (Waltham, MA) and Falcon 40 μm nylon mesh Thermo Fisher Scientific to remove un-disaggregated tissue remnants. The resultant single cell suspension filtrate was used for cell type-enrichment.

### Cell type-specific enrichment

A total of 1.5 ml single cell suspension prepared from fresh prostate tissue or prostate tissue xenografts was mixed with magnetic beads conjugated with an antibody specific for the human endothelial cell surface marker CD31 (25μl of stock solution of 4x 10^8^ beads per ml) from Thermo Fisher Scientific, and incubated at 4°C for 30 minutes. The magnetic beads were “pulled down” using a magnetic stand from Promega (Madison, WI), and washed 5 times with PBS + 0.1% BSA. The cells enriched in the first immuno-magnetic bead selection were endothelial cells. The remainder of the cell suspension after the CD31 magnetic bead enrichment was mixed with Invitrogen Dynabeads Epithelial Enrich from Thermo Fisher Scientific, magnetic beads that were conjugated with an antibody specific for human EpCAM, an epithelial cell surface marker, following the procedure described for endothelial cell enrichment. Cells enriched in the second immuno-magnetic bead selection were the epithelial cells. The heterogeneous single cell suspension that remained after removal of the endothelial and epithelial cell populations was centrifuged at 420 x g for 5 minutes to collect the “stromal cell” population. The ability to handle small tissue specimens was critical to characterization of primary tissue xenografts where the volume of tissue specimens of individual xenografts was <10 mm^3^. Magnetic bead mediated enrichment rather than fluorescence assisted cell sorting (FACS) allows the handling of small tissue specimens (xenografts) in a much shorter time frame.

### RNA isolation from enriched cell populations

RNA was isolated from each enriched cell population specimen using miRNease Micro kits from Qiagen (Germantown, MD) following the manufacturer’s instructions, with modification. RNA was eluted 3 times, each time with 17 μL RNase-free H_2_O, and all the eluents were combined. DNase treatment was performed using a Turbo DNA-free Kit from Thermo Fisher Scientific. After the DNase treatment, RNA was concentrated using an RNA Clean & Concentrator kit from Zymo Research (Irvine, CA). Quantitative assessment of the total RNA yield was performed using the Qubit Broad Range RNA kit from Thermo Fisher Scientific. Before RNA-Seq analysis, the integrity of the RNA was evaluated using a 2100 Bioanalyzer and RNA 6000 Pico kits from Agilent Technologies (Santa Clara, CA). RNA samples accepted for RNA-Seq analysis had RIN values in the range of 6.3–8.

### RNA-Seq analyses

Sequencing libraries for each specimen were prepared from 100 ng of total RNA using the TruSeq Stranded Total RNA kit from Illumina Inc. (San Diego, CA), following manufacturer’s instructions. Ribosomal RNA (rRNA) was depleted from the total RNA, and the remaining RNA was purified and fragmented. The fragmented RNA was primed for cDNA synthesis and reverse transcribed into a first strand cDNA using random primers. The RNA templates were removed, and replacement DNA strands were synthesized to generate double stranded DNA (dsDNA) with dTTP incorporated in place of UTP, resulting in blunt-ended dsDNA. AMPure XP beads from Beckman Coulter (Brea, CA) were used to recover the dsDNA from the second strand reaction mix. A single ‘A’ nucleotide was then added to the 3’-ends of the blunt fragments. Multiple indexing adapters that contained a single ‘T’ nucleotide on the 3’- end of the adapters were ligated to the ends of the dsDNA, preparing them for hybridization onto a flow cell. Adapter ligated libraries were amplified by PCR, purified using Ampure XP beads, and validated for appropriate size on a 4200 TapeStation D1000 Screentape From Agilent Technologies. The DNA libraries were quantitated using KAPA Biosystems qPCR kits, and a sub-specimen from multiple libraries were pooled at equimolar concentrations, following experimental design criteria. Each pool was denatured and diluted to 16 pM for On-Board Cluster Generation, and were sequenced on a HiSeq2500 sequencer using the appropriate cluster kits and rapid mode SBS reagents for 100-cycle paired-end sequencing, following the manufacturer’s recommended protocol from Illumina Inc.

### General Bioinformatic analysis procedures

First-pass, base-pair quality control (QC) was performed using fastqc (v0.10.1). Spliced alignment was performed using Bowtie (v1.0.1) and TopHat (v2.0.13), allowing a maximum of 1 mismatch per read using a joint genome reference strategy (builds hg19 and mm10 using their respective refSeq UCSC annotations). Uniquely mapping human reads were considered for downstream analysis. Separate lane replicates were merged into a single sample alignment file using MergeSamFiles from Picard (v1.97). Second pass QC was performed using alignment output with RSeQC (v2.6.3) in order to check the abundances of genomic features, junction saturation and gene-body coverage. Since mouse tissue may not be completely separated from the primary tissue xenograft, mouse RNA could contaminate RNA samples prepared from the primary xenografts. An additional QC pass was implemented to include only primary xenograft samples that were minimally contaminated with non-human reads. Read counts were obtained using HTSeq (v0.6.0) with intersection-strict option matching to the sequencing protocol. Samples with fewer than 1.7 M processed human reads were filtered out (S1 Fig). Read counts were also normalized using the following estimations: RPKM, TPM and DESeq2-normalization (using R-3.5.1) [28]. Overall sample expression values were visualized using the variance stabilizing transformation implemented by DESeq2. Heat maps, PCA plots, volcano plots, and Venn diagrams were obtained using the following R-3.5.1 libraries: pheatmap, ggplot2, DESeq2 and VennDiagram. All raw counts and fastq files are available from the GEO database (accession number GSE145641).

### Differential expression analysis

Differential expression analysis was performed between the different experimental groups after accounting for potential technical and biological variability using DESeq2. Up- and down-regulated genes were considered to be significant if: adjusted-*p* values were ≤ 0.05 [BH method] and absolute values of fold of difference were ≥ 2. Volcano plots were generated based on differentially expressed genes; targeted gene signatures were highlighted to show their relationship with the groups compared.

### Pathway analysis

Pathway analysis was performed on each of the three specific cell types using Ingenuity Pathway Analysis (IPA) from Qiagen [29]. Gene lists were selected from differential expression analyses, and were employed in canonical pathway analyses using the expression log ratio setting in the core analysis. Cutoffs were set to adhere to IPA instructions to analyze less than 3000 differentially expressed genes.

### Identification of novel cell-type specific marker genes for human prostate epithelial cells, endothelial cells and stromal cells

Novel cell-type specific genes were identified from differential expression analyses between endothelial cells, epithelial cells and stromal cells. A gene was considered to be cell-type specific if for all pairwise cell comparisons the gene was always significantly over-expressed in a single cell type regardless of the contrasting cell’s type or source. That is, an endothelial specific gene must be significantly differentially expressed in endothelial cells when compared to both other cell-types (epithelial or stromal) from both cell sources (fresh tissue or primary xenograft). Comparisons between the same cell-type (e.g. endothelial cells from fresh tissue vs endothelial cells from xenografts) were not included when selecting novel cell-type specific genes.

### Literature-based compilation of lists of cell type-specific genes

“Consensus” epithelial cell- or endothelial cell-specific genes were identified using literature searches for epithelial cell or endothelial cell markers, independent of tissue of origin, that were reported in peer-reviewed journals and had been studied using commercially available antibodies. Lists of 42 epithelial cell-specific genes and 42 endothelial cell-specific genes were compiled. The “consensus” genes and corresponding publications are presented in the (S1 & S2 Tables, and a list of supplementary references).

### Statistics

All statistical analyses in the present study were related to differential expression and pathway analysis and were performed using default software options. p-value or adjusted p-value of ≤ 0.05 or 0.01 were used to indicate statistical significance, as stated in corresponding methods and results.

## Results

### Transcriptomes clustered by cell-type of origin

**Fig 1:**
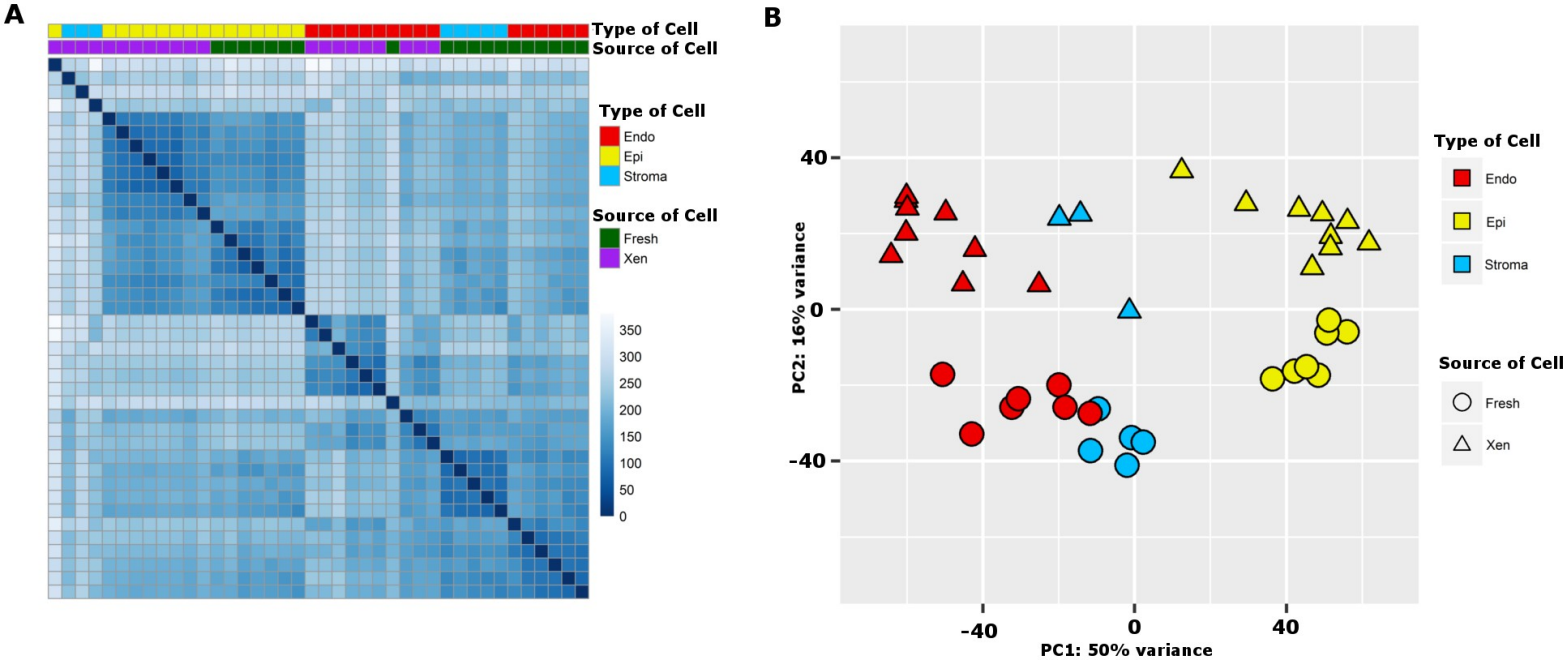
Unsupervised clustering. Un-supervised clustering of transcriptomes of endothelial cells (Endo), epithelial cells (Epi), and stromal cells (Stroma) cells isolated from fresh prostate tissue (Fresh) and primary prostate tissue xenografts (Xen). (A) Un-supervised clustering based on Euclidian distances between sample transcriptomes with samples that are more alike in overall transcriptome being grouped together. Distances scaled from 0 to 350 with darker blue indicating less distance. (B) Principle component analysis of the 40 individual cell type samples from the two tissue sources.

A total of 44 samples of bead-enriched, cell type-specific cell isolates were included in the transcriptome analyses performed with RNA-Seq. The cell-type enriched samples (3 cell types) were prepared from 7 individual fresh prostate tissue specimens procured from 7 patients, and from 11 sets of tissue xenografts established from 11 patients: the patient set from which the xenografts were established did not overlap with the patient set from which fresh tissue was procured. A detailed description of the samples is presented in S3 Table. RNA-Seq data from tissue xenograft specimens were quality controlled to exclude from further data analyses samples significantly contaminated with mouse transcriptomes. Further, xenograft-originated cell type-specific samples that contained less than 1.7 million processed reads were excluded; this resulted in removal of 4 of the total 25 primary tissue xenograft samples. Lastly, all mouse reads included in the individual cell type specific data sets were removed from samples before differential expression analysis. An un-supervised clustering analysis of sample-to-sample differences/similarities between the transcriptomes of the 40 specimens is presented as a heat map (Fig 1A). Transcriptomes of epithelial cells from fresh prostate tissue and from primary tissue xenografts clustered together. Similarly, transcriptomes of endothelial cells from fresh prostate tissue and from primary tissue xenografts clustered together, but separately from either epithelial cell population. Lastly, transcriptomes of stromal cells clustered together in two groups reflecting the tissue specimen type of origin, and clustered separately from transcriptomes of endothelial cells or epithelial cells. Principal component analyses (PCA) of the transcriptomes of all samples demonstrated that endothelial cells and epithelial cells clustered in 2 distinctive groups, whereas, stromal cells formed a third group that was more similar to endothelial cells than epithelial cells (Fig 1B). The variance caused by cell type (50%) was larger than the variance caused by tissue specimen of origin (15%, fresh prostate tissue vs tissue xenografts). The clustering of transcriptomes as three separate cell types validated the experimental enrichment protocol.

### Consensus cell type-specific marker genes were expressed differentially in endothelial, epithelial, and stromal cell fractions

**Fig 2:**
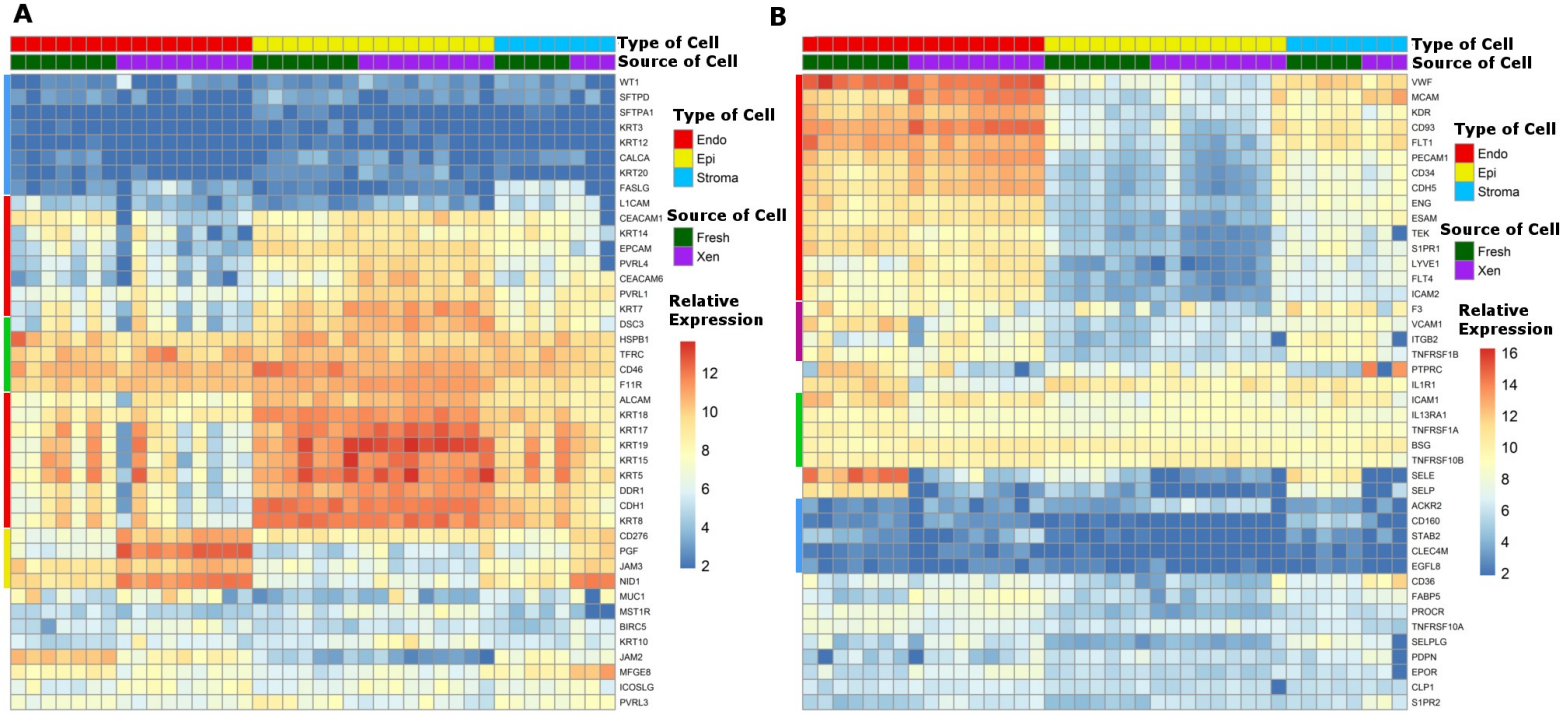
Consensus marker expression. Relative expression of literature-based “consensus” **e**pithelial cell-specific marker genes (A) and endothelial cell-specific marker genes (B) in the different human prostate cell types. Type of cells included: endothelial cells (Endo), epithelial cells (Epi), and stromal cells (Stroma) isolated from fresh prostate tissue (Fresh) and primary prostate tissue xenografts (Xen). The relative expression scale indicates the normalized log_2_ expression level of each gene in each sample. Colored bars on the left of each panel indicate patterns of expression as described in the text.

Expression of consensus “epithelial cell-specific”, “endothelial cell-specific” and “stroma-specific” marker genes identified from the literature (described in Materials and Methods) was compared between the three prostate cell types (Fig 2). Among 42 consensus epithelial cell-specific marker genes, 17 genes (40.5%) were expressed at higher levels in human prostate epithelial cells than in prostate endothelial cells or prostate stromal cells; 4 genes (9.5%) were expressed in all three cell types (base mean > 1100), 9 genes (21.4%) were not expressed or were expressed at very low levels in all three cell types (base mean ≤ 20), 4 genes (9.5%) were expressed at a greater level in endothelial cells than in epithelial cells, and 8 genes (19.0%) were expressed randomly at low levels in a cell type-independent manner (base mean 40~350) (Fig 2A). Since the list of consensus “epithelial cell-specific” genes were selected from literature that spanned a diversity of tissue models and cultured cell lines, it is not unreasonable that this gene set was not highly predictive of genes specific to differentiated human prostate epithelial cells; however, many of the genes clearly are over-expressed specifically by epithelial cells from both fresh prostate tissue and tissue xenografts (Fig 2A). EpCAM, the cell surface protein used for epithelial cell-specific enrichment using antibody-conjugated magnetic beads, was expressed at 12-fold higher levels in prostate epithelial cells than in prostate endothelial cells, supporting this mechanism for high-efficiency enrichment of prostate epithelial cells. The names and the expression levels (base mean) of the “consensus” epithelial cell-specific genes are presented in S1 Table.

Similarly, 42 consensus “endothelial cell-specific” marker genes were identified from the literature: 15 genes (35.7%) were expressed predominantly in prostate endothelial cells compared to prostate epithelial cells; 5 genes (11.9%) were not expressed or were expressed at very low levels in all three human prostate cell types (base mean ≤ 15); 5 genes (11.9%) were expressed similarly among the three prostate cell types (base mean > 450–1350); 4 genes (9.5%) were expressed at similar levels in endothelial cells and stromal cells, but at higher levels in both cell types compared to epithelial cells; and 13 genes (30.9%) were expressed in a cell type-independent pattern (Fig 2B). The names and the expression levels (base mean) of the genes are presented in S2 Table. In contrast to the markers identified for epithelial cells, the consensus markers for endothelial cells were anticipated to be better conserved between endothelial cell populations from diverse organs or cultured cell lines. CD31 (also named PECAM1), the surface protein used for endothelial cell-specific enrichment using antibody-conjugated magnetic beads, was expressed at 59-fold higher levels in prostate endothelial cells than in prostate epithelial cells, supporting the technical approach for enrichment of prostate endothelial cells.

Expression of some consensus endothelial cell marker genes or epithelial cell marker genes was observed in prostate stromal cell populations. Further, the expression profile of the consensus stromal cell type-specific marker genes was more similar to that of prostate endothelial cells than prostate epithelial cells.

### Identification of human prostate endothelial cell-, epithelial cell-, and stromal cell-specific genes from the RNA-Seq data

A total of 176, 48, and 46 cell type-specific genes were identified for human prostate endothelial cells, epithelial cells, and stromal cells, respectively. A gene was identified as specific to a prostate cell type if it was more-highly expressed (fold of difference ≥ 2 with adjusted-*p* value ≤ 0.05) in that cell type compared to both of the other cell types, in both fresh tissue and primary tissue xenograft specimens. Importantly, a selected gene may be expressed in one or both of the other prostate cell types, however, it was expressed at levels at least 2-fold lower than in the index cell type. A detailed list of the human prostate cell type-specific genes identified in the RNA-Seq data is presented in S4 Table. As anticipated, among the 15 consensus endothelial cell markers culled from the literature and confirmed to be human prostate endothelial cell predominant in our RNA-Seq data (presented in Fig 2B), 11 also were identified as differentially expressed in human prostate endothelial cells from both fresh tissue and primary tissue xenografts in our RNA-Seq analysis. In contrast, few of the consensus epithelial cell marker genes from the literature were identified as differentially expressed in prostate epithelial cells.

**Fig 3:**
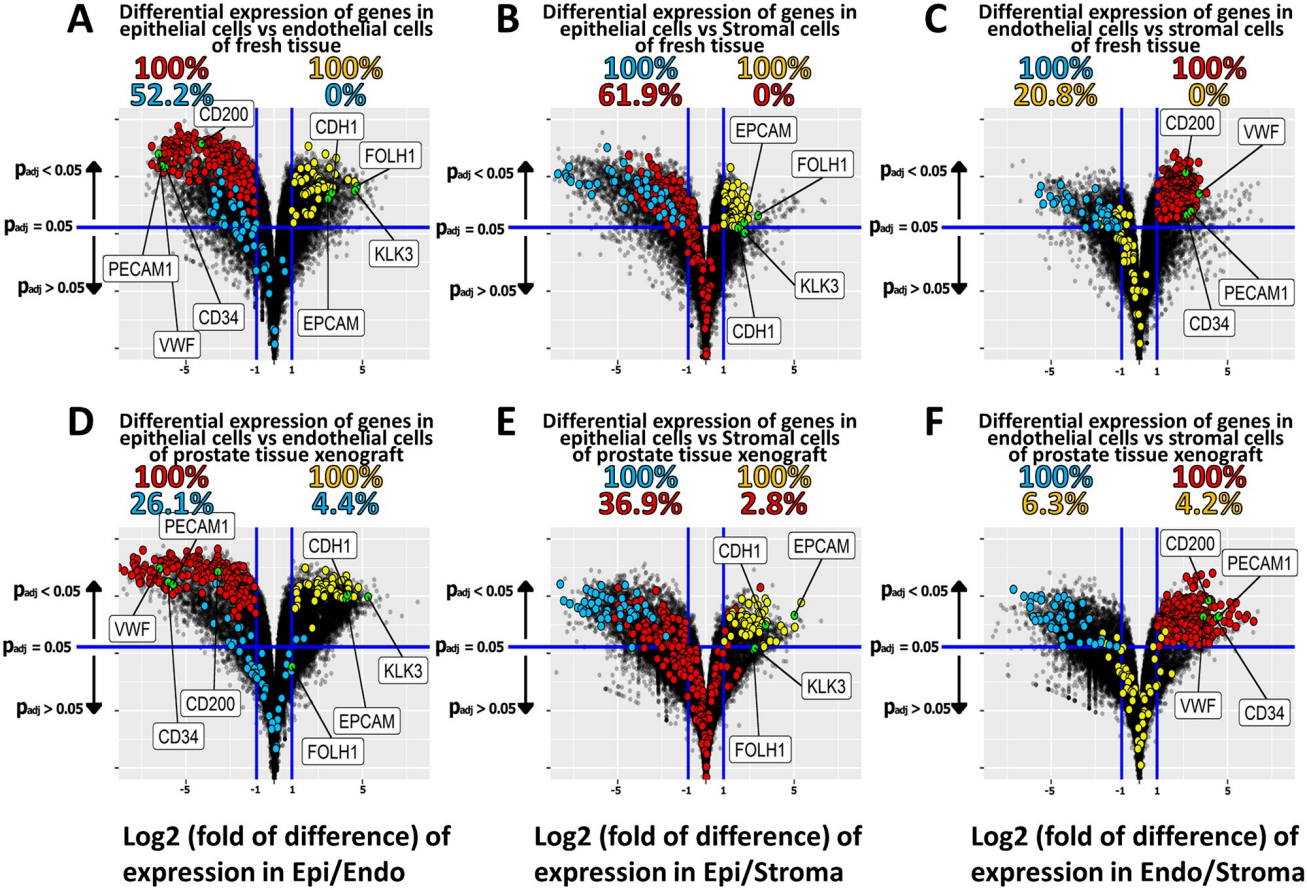
Volcano plot of cell type specific genes. Volcano plot presentation of differential expression of two-way comparisons between cell types isolated from fresh prostate tissue (A-C) and from primary prostate tissue xenografts (D-F). Cell type specific genes identified in this study are highlighted in red, yellow, and blue for endothelial cell-specific (Endo), epithelial cell-specific (Epi), and stromal cell-specific (Stroma), respectively. The horizontal line indicates an adjusted *p*-value of 0.05. An upward arrow indicates a decrease in adjusted *p*-value while a downward arrow indicates an increase in adjusted *p*-value. The two vertical lines indicate a threshold of log_2_ fold of difference of -1 (left) or +1 (right). Genes are considered significantly differentially expressed if they lie on or above the horizontal line with log_2_ fold of difference ≤ -1or log_2_ fold of difference ≥ 1. Percentage values indicate the percentage of the subset of identified cell type specific genes of a particular cell type that were significantly differentially expressed relative to the total number of cell type specific genes for that cell type for both positive log-fold of difference and negative log-fold of difference, on the right and left respectively. In comparisons of endothelial cells the genes CD31 (PECAM), CD34, VWF, and CD200 are indicated while in epithelial comparisons EPCAM, PSA (KLK3), PSMA (FOLH1), and CDH1are indicated.

Two-way comparisons of the complete transcriptomes of prostate epithelial cells versus prostate endothelial cells, epithelial cells versus stromal cells, and endothelial cells versus stromal cells are represented as volcano plots (Fig 3A–3F) to demonstrate visually the extent of differential expression of all of the genes of the entire transcriptome in each comparison. Further, the volcano plots allow highlighting the location of each of the selected endothelial cell-specific genes (176 genes), each of the selected epithelial cell-specific genes (48 genes) and each of the selected stromal-specific genes (46 genes) in all of the 2-way comparisons. This unique strength of volcano plots allows the visual presentation of the differential expression of not only the genes specific for the two cell types being compared, but also genes specific for the third cell type not analyzed in the 2-way plot. In the comparison of epithelial cells isolated from fresh tissue to endothelial cells isolated from fresh tissue, all the genes identified as epithelial specific were markedly over-expressed in epithelial cells relative to endothelial cells, while the endothelial cell- and stromal cell-specific genes were under-expressed in epithelial cells (Fig 3A). Among genes identified as stromal cell-specific, 52% were expressed at higher levels in endothelial cells than in epithelial cells in the comparison of prostate epithelial-endothelial cells (Fig 3A).

Similarly, all stromal cell-specific genes were differentially expressed in stromal cells in the comparison between stromal cells and endothelial cells (Fig 3C). The complete differential-expression of endothelial specific and stromal-specific markers in Fig3C demonstrated that the concordant under-expression of the endothelial and stromal cell specific markers in Fig 3A&3B was not due to the fact that the markers selected as endothelial cell and stromal cell specific have overlapping cell-type specificity.

Two-way comparisons between the three cell types isolated from primary human prostate tissue xenografts (Fig 3D–3F) demonstrated similar patterns as the same cell types isolated from fresh prostate tissue (Fig 3A–3C). The cell type specific markers for prostate epithelial and endothelial cells were segregated effectively in all three two-way comparisons, and the stromal specific markers also were markedly under-expressed relative to the index cell type of the comparison. As observed in the analyses of the cell types isolated from fresh tissue, the marker genes selected as specific for each of the three cell types were up-regulated only in that cell type. The differential expression of epithelial-specific markers EpCAM, PSA (KLK3), PSMA (FOLH1), and CDH1 and endothelial-specific markers CD31 (PECAM), CD34, VWF, and CD200, were identified in the Volcano analyses presented in Fig 3 to present visually the extent of their differential expression. Nonetheless, two conclusions can be drawn from these comparisons: 1) the entire transcriptomes of endothelial cells and stromal cells share some commonality, but both share limited commonality with the transcriptome of epithelial cells; and 2) the cell separation/enrichment protocol was robust, and the heterogeneous “stromal” population had limited contamination with epithelial or endothelial cells.

### Epithelial, endothelial, and stromal cells in tissue xenografts maintained the cell type-specific gene expression signatures of these cells in fresh tissue

**Fig 4:**
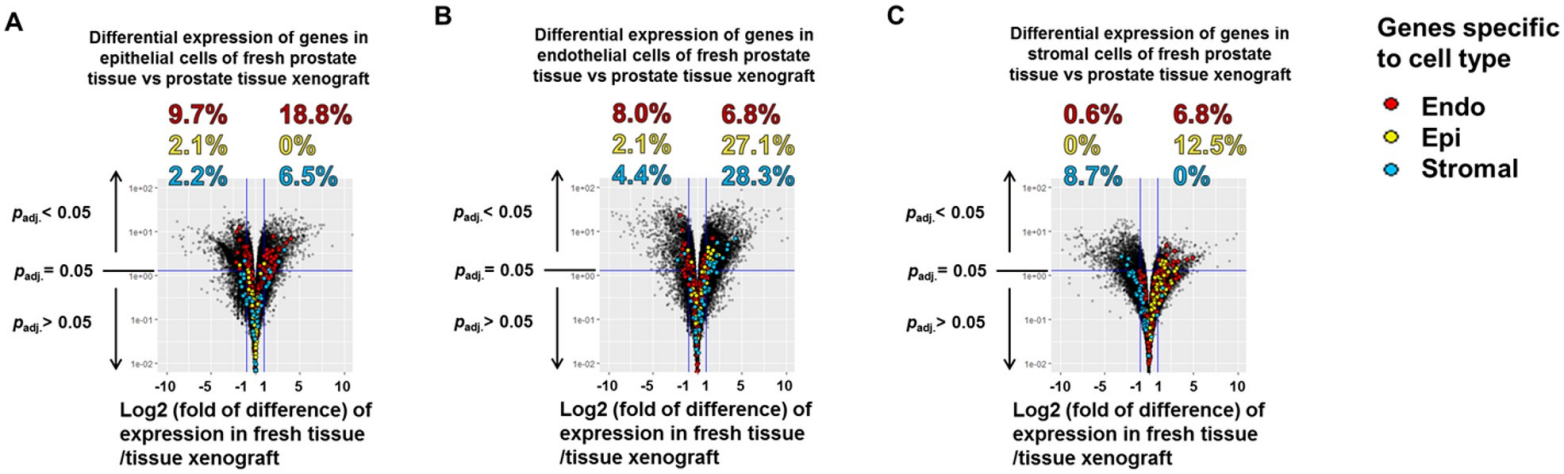
Volcano plot comparison between tissue sources. Volcano plot presentation of differential expression of two-way comparisons between the two tissue sources for epithelial cells (A), endothelial cells (B), and stromal cells (C). All cell type specific genes are highlighted in red, yellow or blue for endothelial cells (Endo), epithelial cells (Epi), and stromal cells (Stroma), respectively. The horizontal line indicates an adjusted *p*-value of 0.05. An upward arrow indicates a decrease in adjusted *p*-value while a downward arrow indicates an increase in adjusted *p*-value. The two vertical lines indicate a threshold of log_2_ fold of difference of -1 (left) or +1 (right). Genes are considered significantly differentially expressed if they lie on or above the horizontal line with log_2_ fold of difference ≤ -1or log_2_ fold of difference ≥ 1. Percentage values indicate the percentage of the subset of identified cell type specific genes of a particular cell type that were significantly differentially expressed relative to the total number of cell type specific genes for that cell type for both positive log-fold of difference and negative log-fold of difference, on the right and left respectively.

Total transcriptome expression profiles were compared for the same cell type isolated from fresh prostate tissue versus from primary xenografts of prostate tissue (Fig 4A–4C). Volcano plots of the 2-way comparisons demonstrate that for each specific cell type a larger fraction of epithelial-, endothelial- and stromal-specific genes were preferentially over-expressed in cells isolated from primary xenografts compared to the same cell type isolated from fresh tissue (Fig 4A, 4B & 4C; boxes). However, only 2% of all the selected epithelial cell-specific genes were differentially expressed between human prostate epithelial cells isolated from fresh tissue compared to isolated from primary xenografts of human prostate tissue maintained on a “humanized” mouse host for 30 days, indicating the *in situ* prostate epithelial cell gene expression signature is highly conserved in the primary prostate tissue xenografts (box, Fig 4A). In contrast, 14.8% of human prostate endothelial cell-specific markers were differentially expressed between human prostate endothelial cells isolated from fresh tissue and endothelial cells isolated from primary xenografts, with 8% and 6.8% of marker genes expressed at higher levels in endothelial cells from primary xenografts or endothelial cells from fresh tissue, respectively (box, Fig 4B). This larger difference in expression profiles between endothelial cells isolated from the two sources compared to between epithelial cells isolated from the two tissue sources could reflect that endothelial cells in primary prostate xenografts are more proliferatively active than endothelial cells in adult prostate tissue *in situ*, or, alternatively, prostate endothelial cells in the xenografts may be responsive to a signal present in the mouse hots microenvironment. Importantly, Fig 4A & 4B demonstrate clearly that the vast majority of the genes identified as human prostate epithelial cell-specific (Fig 4A) and endothelial cell-specific (Fig 4B) were clustered in the region of the volcano plots that indicated no significant difference in expression between cells isolated from fresh tissue and cells from primary xenografts, validating the conservation of the *in situ* transcriptome in the tissue xenografts. Further, the majority of the cell type-specific genes demonstrated very low fold-differences in expression between cells of fresh tissue and tissue xenograft origin, and the level of confidence of their observed differential expression is low compared to the inter-cell type comparisons presented in Fig 3.

### Differential activation of signaling pathways in epithelial, endothelial, and stromal cells

**Fig 5:**
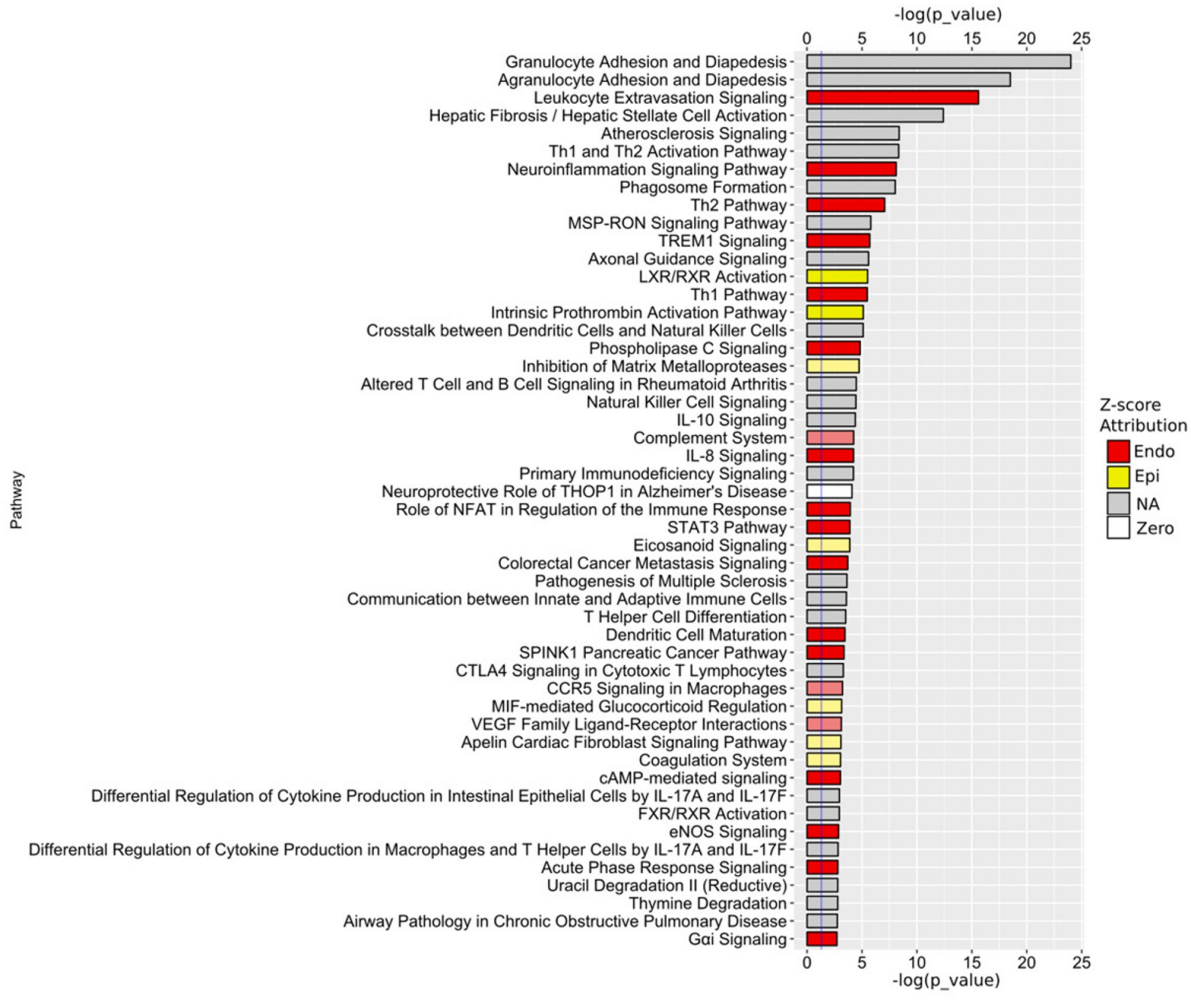
IPA canonical pathways. IPA canonical pathway analysis of epithelial cells versus endothelial cells. Pathways are identified as: more active in endothelial cells (Endo), more active in epithelial cells (Epi), not determined due to insufficient knowledge (NA), or not determined due to insufficient input (zero). The vertical blue line indicates a *p*-value of 0.05 with -log_10_(*p*-value) to the right of the line indicating smaller *p*-value.

**Fig 6:**
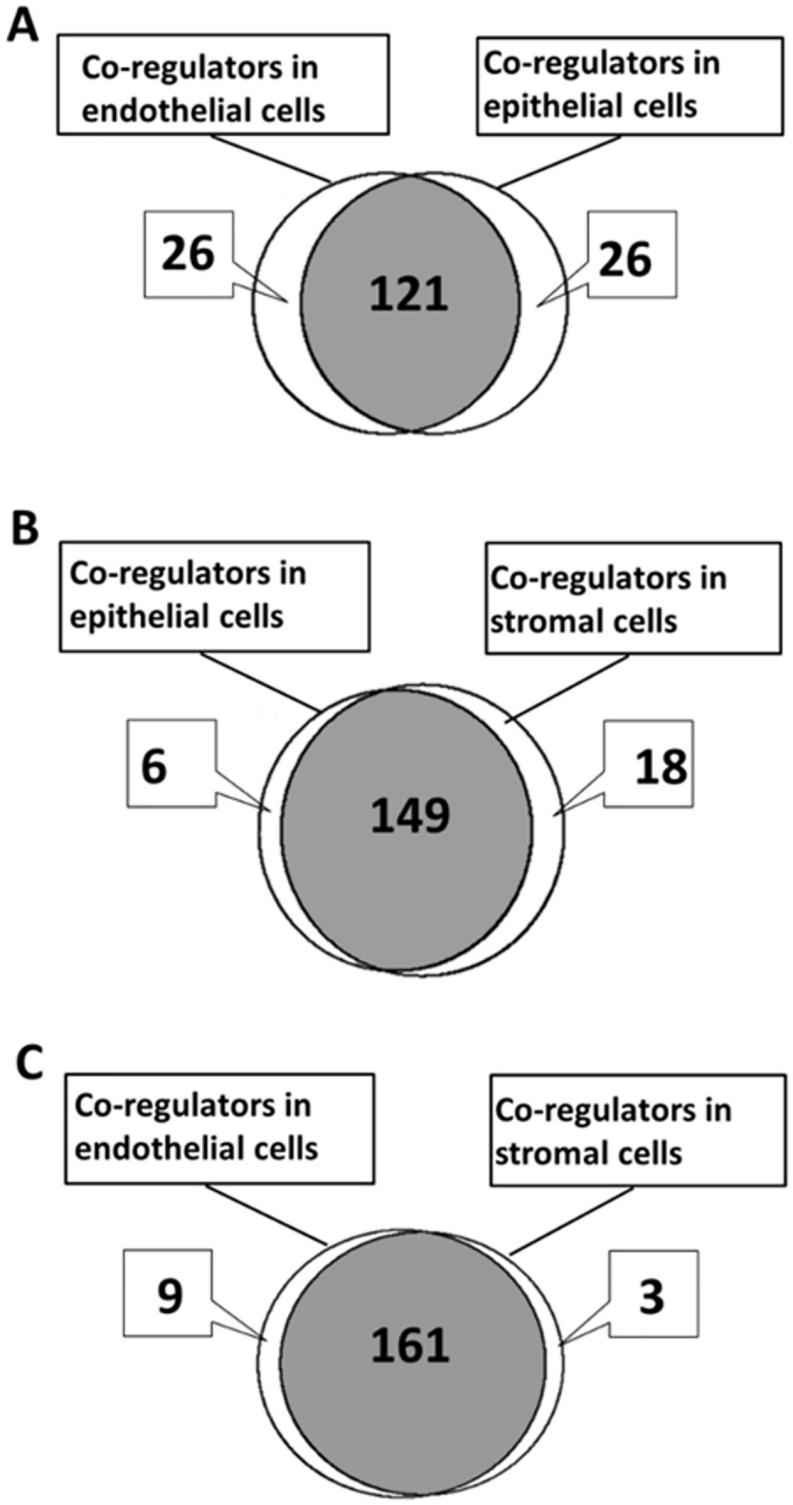
AR co-regulator expression. Venn diagram of differential expression of 173 AR co-regulator genes between endothelial cells vs epithelial cells (A), epithelial cells vs stromal cells (B), and endothelial cells vs stromal cells (C).

RNA-Seq data of epithelial cells, endothelial cells, or stromal cells from both fresh tissue specimens and from primary tissue xenografts were combined as the cell type-specific specimens. Differential expression analyses were performed for epithelial cells versus endothelial cells, epithelial cells versus stromal cells, and endothelial cells versus stromal cells. Genes that were used as input for IPA canonical pathway analyses were selected from these differential analyses based on the criteria of: 1) adjusted-*p* values ≤ 0.05; and 2) log_2_ fold of difference (LFD) ≥ 3 or ≤ -3 in the comparison of epithelial cells versus endothelial cells, LFD ≥ 2 or ≤ -3 in the comparison of epithelial cells versus stromal cells, or LFD ≥ 2 or ≤ -2 in the comparison of endothelial cells versus stromal cells. The selection criteria were chosen to provide the maximum allowed numbers of input genes (≤ 3000, according to instructions of IPA) and similar numbers of genes for each comparison. A total of 1828, 1699, and 1598 genes were selected for IPA analyses from the comparisons of epithelial cells versus endothelial cells, epithelial cells versus stromal cells, and endothelial cells versus stromal cells, respectively. Pathways identified with *p* values ≤ 0.05 in each comparison were considered to be significantly different between the two cell types. The relative “activity” of a pathway in a comparison between two cell types (A and B) was estimated by a “Z” score. In these comparisons a Z score ≥ 2 indicated that a pathway was more active in cell type A than in cell type B, and a positive Z score < 2 indicated that a pathway was likely (or only moderately) more active in cell type A than in cell type B. A Z score ≤ -2 indicated that a pathway was inhibited (or less active) in cell type A than in cell type B, and a Z score between -2 and 0 indicated that a pathway was likely (or moderately) inhibited (or less active) in cell type A relative to cell type B. Therefore, for the purpose of clarity of data presentation, if in a comparison between cell type A and cell type B a positive Z score was reported for a pathway, the pathway was considered more active in A, whereas, if a negative Z score was reported for a pathway, the pathway was considered more active in B. Further, IPA analyses also identified pathways that were significantly enriched in both of the two cell types being compared, but for which the relative activity status could not be determined due to a lack of sufficient “knowledge”, resulting in a Z score of 0, or no Z score. The results of the IPA analyses are summarized in Table 1. A detailed report of the results including the pathways, Z scores, and *p* values are provided in S5 Table. A total of 116, 140, and 96 pathways were found to be significantly enriched when comparing LFD between epithelial and endothelial cells, epithelial and stromal cells, and endothelial and stromal cells, respectively. There were 3 pathways and 1 pathway that were more active (Z scores ≥ 2) in epithelial cells compared to endothelial cells and stromal cells, respectively. In addition, there were 10 pathways and 2 pathways that were likely (or only moderately) more active (positive Z scores < 2) in epithelial cells compared to endothelial cells and stromal cells, respectively. In endothelial cells, there were 37 pathways and 1 pathway that were more active in endothelial cells than in epithelial cells and stromal cells, respectively; and 15 pathways and 9 pathways that were likely or moderately more active in endothelial cells than in epithelial cells and stromal cells, respectively. In stromal cells, there were 63 pathways and 20 pathways that were more active in stromal cells than in epithelial cells and endothelial cells, respectively; and 17 pathways and 26 pathways that were likely or moderately more active in stromal cells than in epithelial cells and endothelial cells, respectively. The IPS analysis revealed an intriguing phenomenon: stromal cells contained the highest numbers of active pathways, followed by endothelial cells, whereas, there were few pathways active predominantly in epithelial cells. This observation suggests that stromal cells and endothelial cells in human prostate are more functionally diverse and dynamic than epithelial cells, whereas, the epithelial cell population is more homogeneous with a limited scope of active functions. The top 50 pathways that were differentially active between epithelial cells and endothelial cells are presented to demonstrate the cell type specificity of the active pathways and the relative differential activity between cell types (Fig 5). The activity of the individual pathways in the comparison of epithelial to endothelial cells are presented in Fig 5 as–log_10_(*p* value) and ranked from largest to smallest: in this log transformation, the actual *p* values are ranked from smaller (longer bars) to larger (shorter bars). All *p* values were ≤ 0.05. Red bars indicate pathways more active (Z score ≥ 2) in endothelial cells, and bars in light hues of red indicate pathways likely or moderately more active (0 < Z score < 2) in endothelial cells. Yellow bars indicate pathways more active (Z score ≥ 2) in epithelial cells, and bars in light hues of yellow indicate pathways likely or moderately more active (0 < Z score < 2) in epithelial cells. Gray or white bars indicate pathways that were different significantly between the cell types, but for which a relative activity status was undetermined. Well characterized pathways associated with endothelial cells, such as the eNOS signaling pathway and the VEGF ligand-receptor interaction pathway, were more active in endothelial cells. In contrast, the androgen signaling pathway was not identified as statistically significant in comparisons between any two cell types. This is consistent with the observation that over 50% of AR-regulated genes are similarly expressed among the three cell types (Fig 6). Further, the IPA results revealed marked activity of a family of prostatic signaling networks implicated in cross-talk with immune cells, and suggest a constant and active interaction of prostate tissue with the immune system. The IPA analysis also suggests that endothelial cells, epithelial cells and stromal cells interact differently with the immune system, even if using the same pathway. The top 50 pathways in comparisons between epithelial cells and stromal cells, and between endothelial cells and stromal cells are presented in S2 and S3 Figs.

**Table 1:** Summary of active pathways in individual cell types identified by IPA analysis.

Comparison between cell Types	Number of Identified Pathways								
	Total	More active in cell type (Z ≥ 2)			Likely/moderately more active in cell type (0 < Z < 2)			Activity status not determined	
		Epithelial Cells	Endothelial Cells	Stromal Cells	Epithelial Cells	Endothelial Cells	Stromal Cells	Z score = 0	No Z score
Epithelial vs Endothelial	116	3	37	-	10	15	-	1	50
Epithelial vs Stromal	140	1	-	63	2	-	17	2	55
Endothelial vs Stromal	96	-	1	20	-	9	26	5	35

### Differential expression of AR co-regulators in epithelial, endothelial, and stromal cells

Among 181 AR co-regulators identified from the literature, 8 co-regulators were expressed at very low levels, or were not expressed, across all samples (base mean ≤ 15); therefore, 173 were selected for evaluation of expression in the three human prostate cell types (Fig 6). Cell type-specific expression of a co-regulator was defined as a fold of difference ≥ 2 and adjusted-*p* ≤ 0.05 in comparisons of differential expression between two cell types. Prostate epithelial cells and endothelial cells shared expression of 121 AR co-regulators that were expressed at similar levels in both cell types. A total of 52 co-regulators were expressed differentially between the cell types, with 26 expressed at ≥ 2-fold higher levels in prostate epithelial cells compared to prostate endothelial cells, while the other 26 were expressed at ≥ 2-fold higher levels in prostate endothelial cells compared to prostate epithelial cells (Fig 6A).

Stromal cells shared expression of 149 co-regulators with epithelial cells; 18 co-regulators were expressed at ≥ 2-fold higher levels in stromal cells compared to epithelial cells, and 6 co-regulators were expressed at ≥ 2-fold greater levels in epithelial cells compared to stromal cells (Fig 6B). Similarly, stromal cells shared expression of 161 co-regulators with endothelial cells, while 3 co-regulators were expressed at least at ≥ 2-fold greater levels in stromal cells than in endothelial cells and 9 co-regulators were expressed at ≥ 2-fold higher levels in endothelial cells than in stromal cells (Fig 6C). The gene names and base means of the differentially and commonly expressed co-regulators identified in these comparisons are presented in (S6 Table). These data suggest the intriguing possibility that AR trans-regulates different spectrums of target genes in different cell types/tissues in response to the availability of different co-regulators. Four co-regulator genes (BAG1, FHL2, MACROD1, TRIM68) were in common between the 26 co-regulators predominantly expressed in epithelial cells compared with endothelial cells, and the 6 co-regulators predominantly expressed in epithelial cells compared to stromal cells (Fig 6A and 6B). These co-regulators represent a conservative list of epithelial cell-specific AR co-regulators in human prostate. Four co-regulators (CAV1, HDAC7, HEY1, RB1) were shared in common between the 26 co-regulators predominantly expressed in endothelial cells compared to epithelial cells and the 6 co-regulators predominantly expressed in endothelial cells compared to stromal cells (Fig 6A and 6C). The 4 co-regulators represent a conservative list of endothelial cell-specific AR co-regulators in prostate. Similarly, 2 co-regulators (GLI2, TAGLN) were in common between the 18 co-regulators predominantly expressed in stromal cells compared with epithelial cells, and the 3 co-regulators predominantly expressed in stromal cells compared to endothelial cells (Fig 6B and 6C); these co-regulators represent a conservative list of stromal cell-specific AR co-regulators in prostate.

### Differential expression of AR-regulated genes in prostate epithelial, endothelial, and stromal cells

**Fig 7:**
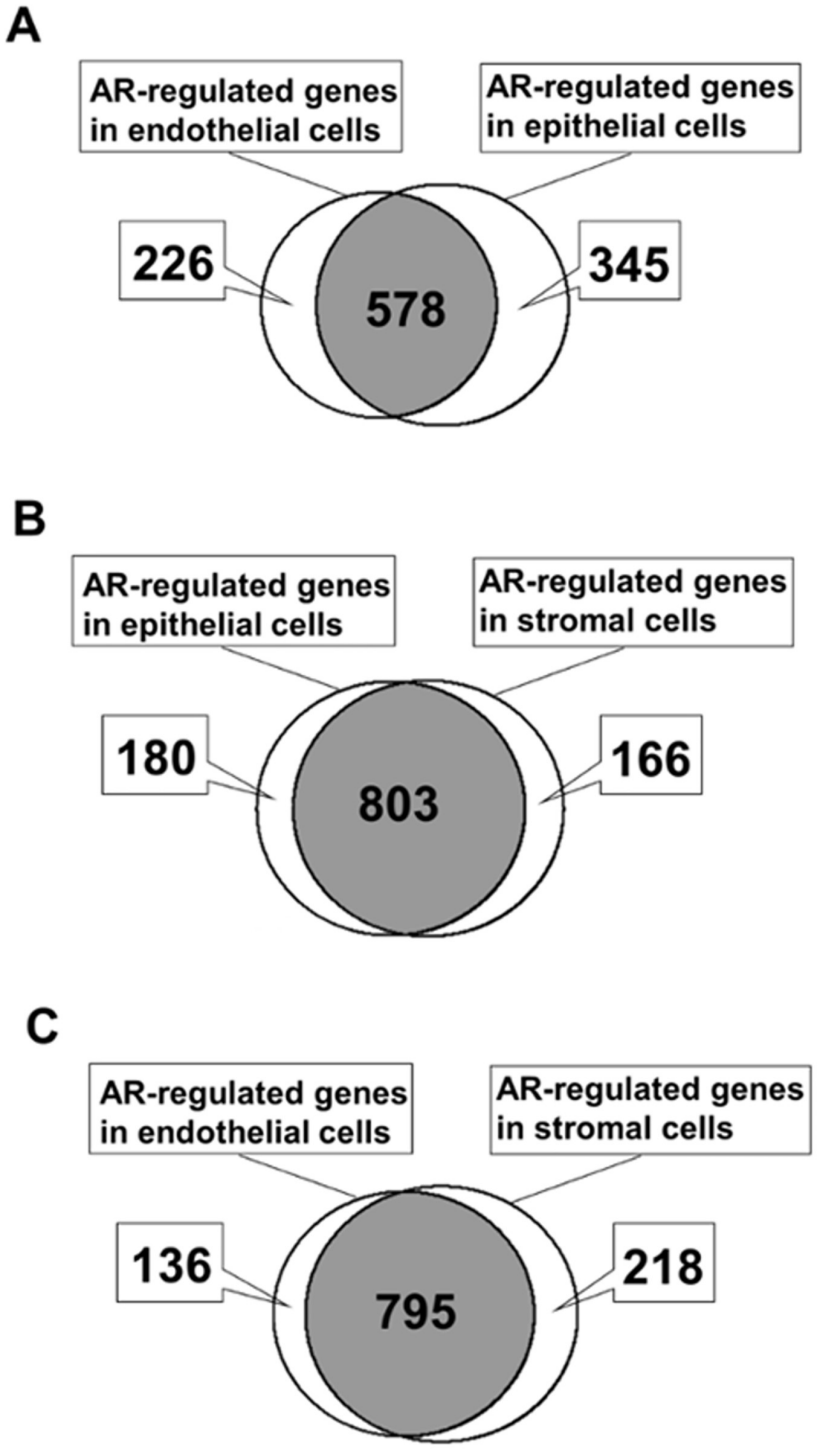
AR-regulated gene expression. Venn diagram of differential expression of 1149 AR-regulated genes between endothelial cells vs epithelial cells (A), epithelial cells vs stromal cells (B), and endothelial cells vs stromal cells (C).

Differential expression of a total of 1149 putative AR-regulated/associated genes that were culled from published works and publicly available databases was evaluated in 2-way comparisons between endothelial cells and epithelial cells, epithelial cells and stromal cells, and endothelial cells and stromal cells (Fig 7). Cell type-specific expression of an AR-regulated gene was defined by a fold-difference in expression of ≥ 2-fold and adjusted-*p* ≤ 0.05 in comparisons between two cell types. Differential expression of AR-regulated genes was most striking in the comparison between human prostate epithelial cells and human prostate endothelial cells, with ~50% of the consensus AR-regulated genes differentially expressed between the 2 cell types (Fig 7A). The differential expression of AR-regulated genes between stromal cells and either epithelial or endothelial cells were of comparable magnitudes (Fig 7B & 7C), but at much reduced numbers compared to the epithelial-endothelial cell comparison. The patterns of differential expression of AR-regulated genes between the human prostate cell types were consistent with the differential expression of AR-co-regulators between the cell types. Conservatively, 133 AR-regulated genes appeared to be epithelial cell-specific in comparisons with both endothelial cells and with stromal cells (in Fig 7A and 7B), 91 AR-regulated genes appeared to be endothelial cell-specific in comparisons with both epithelial cells and with stromal cells (in Fig 7A and 7C), and 52 AR-regulated genes appeared to be stromal cell-specific in comparisons with both epithelial cells and with endothelial cells (in Fig 7B and 7C). Lists of the cell-type specific AR-regulated genes are available in S7 Table.

### Differential expression of androgen metabolic enzyme genes between prostate cell types

**Fig 8:**
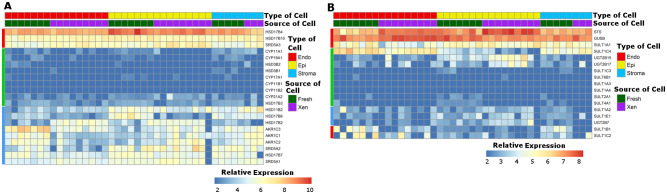
Androgen metabolic enzyme expression. Heat map of relative expression of selected androgen metabolic enzyme genes in endothelial (Endo), epithelial (Epi), and stromal (Stroma) cells. Cells were isolated either from fresh tissue (Fresh) or primary tissue xenograft (Xen). The relative expression scale indicates the normalized log_2_ expression level of each gene in each sample. Key metabolic enzyme genes are presented in Fig 8A. Expression levels of steroid conjugating enzymes, including steroid sulfatases, sulfotransferases, UDP glucuronosyl-transferases and glucuronidases are presented in Fig 8B. Colored bars on the left of each panel indicate patterns of expression as described in the text.

A list of androgen metabolizing/catabolizing genes was compiled based on the conventional knowledge of androgen metabolism and the proposed functions of individual enzymes. The list of genes and relevant references are presented in S8 Table. Expression of consensus androgen metabolizing/catabolizing genes was compared between endothelial cells, epithelial cells, and stromal cells isolated from both fresh human prostate tissue and primary tissue xenografts, and the expression patterns are presented as heat maps in Fig 8A and 8B, respectively. Groups of androgen metabolizing genes are visually linked in the heat maps in 3 distinct categories:1) expressed at comparable levels (base mean > 100) in the 3 cell types (HSD17B4, HSD17B10, HSD17B7, marked by red bar on the left side of the figure); 2) expressed modestly, but differentially, (base mean 25~ 100) in different cell types (AKR1C1/2/3 and SRD5A1/2, marked by blue bar on the left side of the figure); and 3) not expressed or minimally expressed (base mean ≤ 15) in all of the prostate cell types (e.g. CYP17A1, CYP11B1/2 and HSD3B1/2.; marked by green bar on the left side of the figure). Stromal cells shared expression profiles of the metabolizing genes with both endothelial cells and epithelial cells, however, no metabolism genes were expressed predominantly in stromal cells.

Enzymes involved in conjugation and hydrolysis of androgens/androgen metabolites, include sulfotransferases (SULTs) and UDP-glucuronosyltransferases (UGTs), sulfatases (STS) and beta-glucuronidases (GUSB). Differential expression of catabolic genes was evaluated between the three cell types isolated from fresh prostate tissue and primary tissue xenografts (Fig 8B). Genes expressed at relatively high levels in this group (base mean ≥ 30) were marked with a red bar on the left side of the figure. STS, GUSB, and SULT1A1 were expressed similarly in all 3 cell types. SULT1B1 and SULT1C2 were expressed at higher levels in endothelial cells and stromal cells than in epithelial cells. In contrast, UGT2B15 and UGT2B17 were expressed at higher levels in epithelial cells than in endothelial cells or stromal cells. A majority of SULTs, however, were not expressed in any of the 3 cell types in prostate (marked with a green bar on the left side of the figure). Also, there was a subset of androgen catabolic genes (marked with a blue bar on the left side of the figure) that were expressed at relatively low levels (base mean < 3) in the 3 cell types, and did not demonstrate cell type specificity. Importantly, the expression profiles of androgen metabolic/catabolic enzymes were conserved for each individual cell type whether isolated from fresh prostate tissue or from primary tissue xenografts. These cell type specific gene expression profiles should raise caution for interpretations of intracrine (prostate tissue) androgen synthesis. CYP11A7, HSD3B1, and HSD3B2 are key enzymes in T and DHT synthesis by the front-door and back-door pathways [30, 31], however, they were not expressed in any prostate cell type. Further, expression of AKR1C3 (HSD17B5), a key enzyme of the front door pathway, and that has been proposed as an enzyme critical for intracrine synthesis of T/DHT [32, 33], was limited to prostate endothelial cells.

### Differential expression of ATP cassette-binding (ABC) efflux pumps, solute carrier (SLC) transporters, and solute carrier organic anion (SLCO) transporters between different prostate cell types

**Fig 9:**
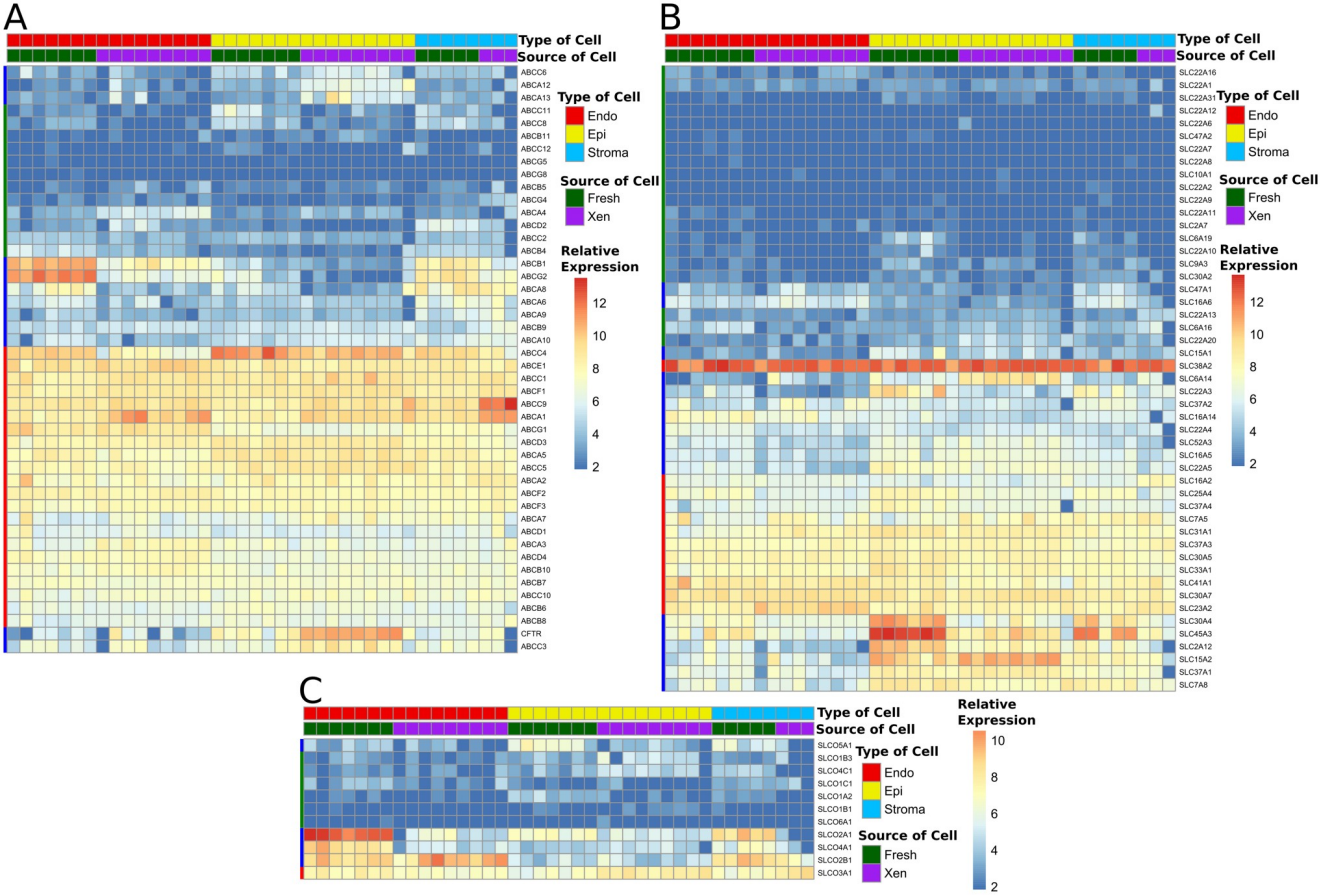
Relative expression of ABC pumps, SLC and SLCO transporters. Heat map of relative expression of selected ATP cassette-binding (ABC) efflux pumps (A), solute carrier (SLC) transporters (B), and solute carrier organic anion (SLCO) transporters (C) in endothelial (Endo), epithelial (Epi), and stromal (Stroma) cell types. Cell types were isolated from fresh tissue (Fresh) or primary tissue xenografts (Xen). The relative expression scale indicates the normalized log_2_ expression level of each gene in each sample. Colored bars on the left of each panel indicate patterns of expression as described in the text.

Expression profiles of 46 ABC efflux pumps, 49 SLC, and 11 SLCO were compared between the three prostate cell types (Fig 9). ABC pumps function to efflux therapeutic drugs and conjugated steroids out of cells and, therefore, to affect drug efficacy and hormone levels in cells [34, 35]. SLC transporters are responsible for uptake of a variety of substances, including nutrients, ions, anions and therapeutic drugs [36]. SLCO transporters are organic anion transporters that uptake therapeutic drugs and steroids/steroid conjugates into cells [35, 37, 38]. Detailed description of the selected genes and relevant references are presented in S9 Table. Expression profiles of the genes of the three super-families are presented as heat maps generated using normalized expression levels, to reflect relative levels of gene expression in each cell type from each source of tissue. Genes expressed at relatively high levels (base mean ≥ 70) in all three cell types are marked with a red bar on the left side of the figure, genes that were not expressed or are expressed at low levels (base mean < 15) in all three cell types are marked with a green bar, and genes expressed with cell type specificity are marked with a blue bar. (Fig 9A, 9B and 9C). Among the ABC efflux pumps, ABCC4 and CFTR were expressed predominantly by prostate epithelial cells, while ABCC1 and ABCG2, both potent chemotherapeutic drug and steroid-conjugate efflux mechanisms, were expressed predominantly by endothelial cells (Fig 9A). All three prostate cell types shared expression of many SLC uptake transporters, including SLC38A2 and isotypes of SLC23, 30, 31, 33, 37 and 41. Prostate epithelial cells appeared to differentially express SLC30A4, SLC45A3, SLC2A12, SLC6A14 and SLC15A2, whereas, endothelial and stromal cells did not appear to differentially express any SLC transporter. In contrast, prostate endothelial cells differentially expressed SLCO2A1, 2B1, 3A1 and 4A1 consistent with their role in the uptake of macro-molecules from serum into the tissue. Further, prostate endothelial cells demonstrated a slight differential expression of SLCO5A1.

## Discussions

The goal of these studies was to determine the fidelity with which the individual cell components of primary xenografts of human prostate tissue recapitulate the same cell type in prostate tissue *in situ*. Analysis of the differential expression of cell type-specific genes between the corresponding type of cells enriched from fresh prostate tissue specimens and from fresh prostate tissue xenografts demonstrated conservation of expression of cell type-specific genes in the tissue xenografts, as well as indirectly demonstrating the efficiency of the cell type-specific enrichment. Therefore, this study suggests that primary xenografts of fresh surgical specimens represent the optimal model for study of the role of tissue architecture, inter-cellular communication and differential permeability mediated via the endothelial cells of the tissue microvasculature. Further, the present study at the global transcriptome level validated the utility of cell type-specific enrichment by magnetic beads conjugated to antibodies specific for cell surface proteins. The purity of the enriched endothelial cell fraction and the epithelial cell fraction was confirmed by the differential expression of known endothelial cell or epithelial cell related genes in the bead selected cell populations. The establishment of an efficient protocol for cell type-specific enrichment using surface marker-specific conjugated magnetic beads allowed demonstration at the level of the total transcriptome level that cellular gene expression profiles were maintained in specific cell types in the xenografts. Further, demonstration of the suitability of magnetic bead mediated enrichment rather than fluorescence assisted cell sorting (FACS) allows the handling of small tissue specimens (xenografts) in a much shorter time frame. The ability to handle small tissue specimens was critical to characterization of primary tissue xenografts where the volume of tissue specimens of individual xenografts was <10 mm^3^. Further, the shorter time required for sample processing facilitated maximal preservation of the transcriptomes of the specimens. CD31, along with VWF and CD34 all have been used for isolation of endothelial cells from various tissue types. Recently, CD200 was identified as a new endothelial cell marker in a study using single cell RNA-Seq data of the prostate [39]. The expression of CD31, VWF, CD34, and CD200 was evaluated in our differential expression analyses. CD31, CD34, VWF, and CD200 all were expressed predominantly in the endothelial cell enriched fraction compared to the epithelial cell fraction or stromal cell fraction (Fig 3). CD31 and CD200 behaved comparably in our experimental settings, although CD200, in general, had a lower overall expression level in endothelial cells, and a smaller fold-difference of expression compared to the epithelial and stromal cell fractions. Our present work and the work by other groups all indicate the need for cell type-specific approaches for studies of prostate endothelial cells. All approaches have their merits and limitations. Different methodological approaches should not be viewed as mutually exclusive, and the data obtained using different approaches should not be perceived as potentially contradictory, but rather complementary and cross-supporting.

Previous reports focused on cell type-specific transcriptomes in either epithelial or “stromal” cells, whereas, transcriptome analyses of prostatic endothelial cells *in situ* were not available. Sequential rounds of selection by cell surface marker-conjugated magnetic beads from the enzymatic disaggregate of a single piece of tissue, or a pool of matched xenografts, provided the accurate characterization of the transcriptome of individual cell types, and the most comprehensive comparison of transcriptomes among different cell types from the same specimen, or between specimens from fresh tissue versus from primary xenografts. This approach also provided the most comprehensive characterization of transcriptomes for discovery of new genes, network of genes, and functional signaling pathways specific to human prostatic endothelial, epithelial, and stromal cells. However, conclusions based on the stromal cell transcriptome reported here require a caveat. Unlike the endothelial cell and epithelial cell populations that are positively enriched using cell type-specific surface markers, the “stromal cell” fraction is the remaining pool of cells after sequential selection of endothelial and epithelial cells. The stromal cell fraction contains not only multiple mesenchymal cell types, but also may contain residual endothelial cells or epithelial cells not recovered during the positive selection steps. The complexity of the stromal cell population, and the possible presence of residual endothelial and epithelial cells, is suggested by the partial overlap of gene expression profiles between the stromal cell fraction and endothelial cells and epithelial cells. However, the identification of a set of differentially expressed genes specific to the prostate “stromal” cell population (Figs 3 and 4) suggested that the stromal cell fraction did represent a largely unique cell population from which important transcriptome information could be gleaned. Multiple surface marker antibody-based cell isolation methods need to be developed in order to obtain more decisive transcriptomes of the multiple cell types in the stromal tissue.

There has been a lack of studies that evaluate how much primary tissue xenografts accurately model *in situ* tissue. The present study is the first to test definitively the extent of recapitulation of *in situ* tissue gene expression profiles by primary tissue xenografts. The short-term fresh tissue xenograft model preserved key features of benign prostate tissue morphology *in situ*, for example, the glands were lined with a single layer of columnar epithelial cells, and AR was observed to maintain nuclear localization in the luminal epithelial cells suggesting the AR has bound androgen and is active in trans-regulation of transcription (S4 Fig). Molecular characterization of tissue xenografts demonstrated the preservation of human tissue architecture as well as the maintenance of a human endothelial cell lined micro-vasculature. The data presented in this study provides substantial evidence in support of the hypothesis that primary tissue xenografts represent a valuable and accurate *in vivo* model for prostate tissue *in situ*. The global gene expression profiles of each cell type from fresh tissue specimens were highly preserved in tissue xenografts. Further, genes that are immediately relevant to current prostate cancer research, such as AR-regulated genes, co-regulators of AR-mediated transcriptional regulation, and androgen metabolic/catabolic enzymes were well preserved in primary tissue xenografts, and demonstrated epithelial-, endothelial- and stromal cell-specific patterns of expression. One of the key values of using the short-term, fresh tissue xenograft is that the human vasculature and human endothelial cells are preserved in the tissue, and represent essentially the entire vascular network within the xenograft. Prolonged maintenance results in a gradual invasion of mouse host vasculature at the periphery of xenografts. The trauma of serial passaging results in the replacement of the human endothelium and stroma with cells from the mouse host, negating the value of the model. Differences in the transcriptomes of the same cell type between xenograft and fresh tissue were also noted but not surprising. Multiple factors may contribute to the differences, for instance, the change of host from human to mouse. A significant contribution of our study is that our data provides definitive descriptions for the type and extent of the differences, and furthermore, in a cell type-specific manner. A potential application of our data is to offer evidence for investigators who are interested in using fresh tissue xenograft in order to decide what functions and signaling pathways are preserved or changed in the fresh tissue xenograft, and to what extent, and therefore, what pathways and functions may be studied using the model.

The characterization of cell type-specific transcriptomes allowed identification of sets of genes that are specific to prostate endothelial, epithelial, and stromal cells. These cell type-specific genes are directly relevant to research in basic prostate biology and prostate cancer etiology/progression-. As shown in Figs 4 and 5, “consensus” endothelial, epithelial, or stromal cell-associated genes are not necessarily specific in the respective cells types in prostate. Consequently, the human organ specificity of cell type-specific genes must be identified rather than using a generic list of genes that often were identified in cell culture. This study demonstrates that the vast majority of “consensus” cell type-specific genes were not expressed differentially in the cell types of human prostate. The discrepancies probably reflect differences in the sources of cells, fresh tissue and primary xenografts in this study versus cells maintained in culture that allowed adaptation of the gene expression signature to optimal growth in culture, and also the uniqueness of prostate tissue compared to other tissue or organs profiled in the literature. Further, the complexities on the cell type-specific gene expression profile in vivo clearly affected by the unique cell-cell interactions that only occur in an intact tissue microenvironment.

An intriguing observation made through IPA analysis revealed that multiple immune-related pathways were among the most predominant pathways expressed differentially between the different prostate cell types. In general, the identified immune-related pathways were more active in endothelial cells and stromal cells compared to epithelial cells, a phenomenon that agrees with the function of endothelium as an interface between the prostate tissue and the body, and supports a close interaction between stromal tissue and the immune system. Interestingly, the glucocorticoid receptor signaling pathway, and some pathways that are related to immune cell signaling, wnt signaling, and bone metamorphosis also were identified in a study using the prostate of the TRAMP mouse model in comparison with prostates of non-transgenic littermates [40]. The work using the TRAMP model was focused on the comparison between cancerous and benign mouse prostate, whereas, the current study was focused on the comparison of endothelial cells, epithelial cells, and stromal cells of benign human prostate. Nevertheless, it is of interest to examine further the relevance of the signaling pathways identified in the studies. On the other hand, critical differences between the two models may be responsible for the majority of differences between the findings of the two studies. The most important difference between the tissue xenograft model and the TRAMP model is the origins of the cells. Epithelial cells, endothelial cells, and stromal cells in the primary prostate xenografts are of human origin, whereas, all cell types in the TRAMP model are mouse cells. The current study is focused on benign prostate tissue, whereas, the TRAMP model is used primarily for the study of tumor development. A second issue, is that all of the cells in a TRAMP mouse contain a strong viral transforming gene with a probasin promoter (SV40-Tag oncogene) that could have dramatic effects on the transcriptome that pervert normal gene expression profiles.

AR is a critical mediator of organogenesis, prostate development during puberty and maintenance of prostate structure and function in adults [1, 41]. However, studies of the function of AR in prostate are focused predominantly in epithelial cells. Our previous study demonstrated that castration-induced apoptosis of prostate endothelial cells preceded the apoptosis of prostate epithelial cells, and the response of androgen-responsive promoter activity was different between human prostate endothelial cells and epithelial cells, suggesting different repertoires of AR-trans-regulated genes may be active in the two cell types [20]. In the current study, expression profiles of AR-regulated genes and AR co-regulator genes were compared between human prostate endothelial, epithelial, and stromal cells. Overlapping expression profiles indicated shared AR functions between different cell types, which is not un-expected. However, subsets of both AR-regulated genes and AR co-regulator genes showed cell type-specific patterns of expression. Comparison of gene expression profiles between endothelial cells and epithelial cells identified 26 AR co-regulators that were expressed predominantly in endothelial cells and 26 AR co-regulators that were expressed predominantly in prostate epithelial cells. The most endothelial cell-specific AR co-regulators included: CAV1, a caveolar scaffold protein and AR-activator [42, 43]; HDAC7, a histone deacetylase and AR co-repressor [44]; HEY1, a basic helix-loop-helix transcriptional repressor that acts as an AR co-repressor [45]; and RB1, a tumor suppressor that represses AR expression and AR-regulated transcription via E2F transcription factor 1 [46]. The prostate epithelial-specific AR co-regulators included: BAG1, a regulator of heat shock protein 70, and a long protein isoform (BAG1L) that interacts with and sensitizes AR [47]; FHL2, a selective ligand-dependent co-activator of AR [48, 49]; MACROD1 (also called LRP16), an AR co-activator [50]; and TRIM68, a putative E3 ubiquitin ligase that acts as an AR co-activator [51]. How differences in expression of AR co-regulator genes contribute to the differential expression of AR-regulated genes is under investigation. Historically most AR co-regulators were identified using prostate cancer cell lines, and, thus, were disproportionally or exclusively limited to epithelial cells. Since the cell type-predominant AR co-regulators were identified in this study using DE comparisons between enriched cell populations, predominant expression of a co-regulator in endothelial cells does not necessarily exclude expression in epithelial and/or stromal cells. It is of interest to determine whether differences in the relative expression of AR co-regulators between cell types could contribute to modulation of the transcription profiles of AR-regulated genes in the different cell types. More importantly, our data provide critical information for identification of new AR co-regulators that may be expressed exclusively in endothelial cells. Due to the limitation of availability of quality antibodies specific for the co-regulators, confirmation of the expression of individual co-regulators at the protein levels is lagging. Nevertheless, the data suggests that the cellular context of AR co-regulator expression may play a role in the different AR functions in different cell types. The implication of these findings is that the response to androgen stimulation by benign/malignant prostate tissues, and response to androgen deprivation therapy (ADT) of benign/prostate cancer tissues, needs to be evaluated separately in the different cell types due to the presence of different cell type-specific AR signaling networks. Additionally, the response to androgen stimulation, and to ADT, of a particular cell type needs to consider the role of AR-driven interactions between cell types.

Analysis of the expression of androgen metabolic enzymes revealed the complex nature of the intracrine metabolism of androgens within human prostate tissue. Metabolism of androgen, especially the target tissue production of T or DHT, has been reported in a variety of studies that focused on prostate cancer tissue, and suggested intracrine metabolism as a mechanism for prostate cancer cells to evade the adverse effects of castration [30, 31]. However, these studies did not consider the individual capabilities/contributions of the specific cell types within the prostate. The comparative analysis of this study found that the general pattern of expression of androgen metabolic enzyme genes was similar among the three cell types. While HSD3β1, HSD3β2, and HSD17β3 are key enzymes for the production of T/DHT from DHEA (the front-door pathway), expression of these genes either was not detectable or was at very low levels. Therefore, based on gene expression profiles, prostate tissue appears to have a weak capacity to convert DHEA to T or DHT, an observation that is consistent with our recent report that benign prostate tissue converted DHEA to DHT only if DHEA was present at a supra-physiological concentration of 3.5 μM [25]. Back-door and alternative back-door pathways also were proposed as mechanisms for prostate cancer tissue to produce DHT without production of T as an intermediate [52, 53]. The key enzymes for the back-door and alternative back-door pathways were expressed in prostate tissue: HSD17β6 (predominantly expressed in epithelial and stromal cells); HSD17β10 (universally expressed in all three cell types); and AKR1C3 (expressed in all three cell types, but highest in endothelial cells from fresh prostate tissue) (Fig 8). The expression profiles for these genes suggested a potential for a back-door pathway mediated intracrine production of DHT. However, expression of CYP17A1, the most critical enzyme for intracrine production of DHT via a back-door pathway, was not detectable or was at very low levels, suggesting these metabolic pathways are not functional in prostate tissue. On the other hand, enzymes that were expressed at relatively high levels in human prostate cells that are associated with various aspects of androgen metabolism include HSD17β4, HSD17β2, and AKR1C2) that deactivate T or DHT by conversion T or DHT to inactive metabolites. Further, the expression of enzymes that conjugate androgen metabolites to sulfur or glucuronide to facilitate efflux out of the cells, including SUL1A1 and UGT2B15, also were expressed at relatively high levels. Together the data indicated that androgen metabolic enzymes in prostate tissue are more attuned to deactivation and removal of T and DHT from cells rather than to production of DHT. Prostate tissue in a healthy adult human male has available a constant supply of T and DHT from the circulation. Therefore, it is reasonable that the profile of androgen metabolic enzymes in healthy prostate tissue is focused on removal of T or DHT to maintain a homeostasis of AR activity rather than on generating more T and/or DHT to further stimulate AR activity. Since benign prostate tissue was used in this study, the data do not contradict the hypothesized intra-tumoral production of T/DHT through front-door and/or back-door pathways.

The ATP-cassette binding efflux pumps (ABC pumps) are a large family of trans-membrane pumps that play an important role in the efflux of both conjugates of endogenous polycyclic chemicals and chemotherapeutic drugs and, therefore, are associated with multiple-drug resistance in many types of cancer [34, 54]. Among 46 ABC pumps that were examined for expression, over 50% were expressed in prostate (Fig 9A), with the majority expressed universally across cell types. ABCB1 and ABCG2 were expressed predominantly in endothelial cells, with low levels of expression in epithelial cells. ABCB1, also called drug export pump P-gp, or multi drug resistance 1 (MDR1), belongs to the ABCB family of ABC pumps. ABCB1 is capable of the efflux of multiple anticancer drugs, including docetaxel, etoposide and vincristine. The substrates of ABCG2, also called breast cancer resistance protein (BCRP), include anticancer drugs and steroid conjugates, such as estrone-3-sulfate and dehydroepiandrosterone sulfate (DHEA-S). Although expression of both ABCB1 and ABCG2 are commonly considered only in epithelial cells or cancer cells, the predominant expression of these pumps in prostate is in endothelial cells. Expression in prostate endothelial cells validates their role in regulation of access of steroids/steroids conjugates to prostate tissue, as well as a mechanism of drug resistance in prostate cancer. ABCC4 and CFTR are expressed in all three prostate cell types, but with the highest level of expression in epithelial cells. ABCC4 is also called MRP4. Substrates of ABCC4 include DHEA-S [55, 56], and the expression of ABCC4 in primary prostate cancer tissue was reported to be reduced upon androgen ablation, suggesting a role for AR in regulating expression of ABCC4 [57]. The higher expression of ABCC4 in prostate epithelial cells indicates this ABC pump is important for regulation of accumulation of DHEA-S in epithelial cells. CFTR, also called ABCC7, is a cyclic adenosine mono-phosphaste-regulated anion channel [54, 58] that has been suggested to be a tumor suppressor gene in human intestinal cancer [59]. However, knockdown of CFTR expression in prostate cancer cells enhanced sensitivity to cisplatin of [60]. Expression of ABCC4 and CFTR in prostate epithelial cells suggests that the two ABC pumps may carry unique, prostate epithelial cell specific functions among all ABC pumps.

The super family of SLC transporters is responsible for uptake of a large variety of solute organic ions or organic anions, including nutrients, xenobiotics, and therapeutic drugs [54]. Among the 49 SLC transporters examined (Fig 9B), ~50% were not expressed, or were expressed at very levels, in any of the prostate cell types. SLC38A2 was the only SLC transporter expressed at high levels in all three prostate cell types. SLC38A2 encodes the sodium-coupled neural amino acid transporter 2 (ATA2) whose expression was reported to be highest in heart tissue and the placenta, although prostate tissue was not included in this comparison [61]. Information on the function of SLC38A2 in prostate, and the relative abundance of the transporter in prostate compared to other organs, is not available. SLC45A3, SLC30A4, SLC15A2, and SLC2A12 were expressed at higher levels in prostate epithelial cells compared to endothelial cells. SLC45A3 encodes prostate cancer-associated protein 6 (prostein) [62]. The expression of SLC45A3 was reported to be lower in prostate cancer tissue compared to benign tissue, however, androgen induced expression of SLC45A3 in prostate cancer cells [63]. Gene rearrangements in SLC45A3 were concurrent with ERG rearrangements in a prostate cancer cohort [64], with SLC45A3-ERG gene re-arrangement associated with loss of SLC45A3 protein expression and an unfavorable clinical course in prostate cancers [65]. Further, a genome-wide association study (GWAS) found a single nucleotide polymorphism (SNP) in SLC45A3 was associated with prostate specific antigen (PSA) levels [66]. SLC30A4 encodes Zinc transporter 4 (hZnT-4) [67] that is a potential target of micro RNA MiR-452-5p that has been associated with the development of prostate cancer [68]. Both down-regulation [69] and upregulation of hZnT-4 at the mRNA level [70] was found to be associated with abnormalities in prostate or prostate cancer. Our data demonstrated that expression of SLC30A4 was predominantly evident in prostate epithelial cells, suggesting, the altered expression of the gene in prostate cancer likely is an epithelial cell event. SLC15A2 encodes peptide transporter 2 (PEPT2) [71] that was over-expressed in prostate cancer cell lines [72], however, expression of SLC15A2 at the mRNA level was found to be lower in prostate cancer tissue than benign prostatic hyperplasia (BPH) tissue [73]. Interestingly, a variant of SLC15A2 predicts the severity of porphyria-associated kidney disease [74]. SCL2A12 encodes facilitated glucose transporter member 12 (GLUT12) [75]. In culture, expression of GLUT12 promoted prostate cell growth, mediated AR-regulated glucose uptake, and was regulated by both androgens and calcium/calmodulin-dependent protein kinase kinase 2 (CaMKK2) [76]. SLC16A14 is among the few SLC transporters expressed predominantly in prostate endothelial cells. Although SLC16A14 belongs to the 14-member family of SLC16A that encode mono-carboxylate transporters (MCTs), this particular transporter has no known function. However, higher expression of SLC16A14 was associated with longer progression-free survival of patients with ovarian cancer [77, 78], and, conversely, was implicated in resistance to chemotherapy of ovarian cancer cell lines [79, 80].

Members of the SLCO transporter family are responsible for cellular uptake of a wide range of substrates that include xenobiotics, therapeutic agents, and steroid-sulfate and glucouronide conjugates [35, 37, 38]. There are 11 members of the SLCO transporter family. Although the role of SLCO transporters in normal cells have not been thoroughly studied, some of the SLCO transporters were reported to be important in prostate cancer. Changes in the expression levels of SLCO transporter genes between benign prostate tissues or primary prostate cancer and metastatic or castration-resistant prostate cancer tissues were reported [81]. The chemotherapeutic drugs paclitaxel and docetaxel are known substrates for SLCO1B3 and 1B1 [82, 83]. Similarly, the steroid conjugates dehydroepiandrosterone sulfate (DHEA-S), estrone sulfate (E-S) and estradiol glucuronide are substrates for SLCOs 1A2, 1B1, 1B3 and 2B1 [35, 37, 38]. Since DHEA-S and E-S are substrates for synthesis of other important steroids, including T, DHT and estrogens, the uptake of DHEA-S and E-S may be important to regulation of tissue androgen (and estrogen) homeostasis, affecting AR activity and AR signaling in prostate tissue. Additionally, SLCO transporters are important in the regulation of the availability of systemically applied chemotherapeutic agents to prostate cancer cells. Expression of SLCO transporters in benign prostate tissue and prostate cancer tissue have been examined primarily by analysis of RNA isolated from the whole tissue, therefore, little information is available concerning the cell type-specific expression of these transporters. Our data shows that the transporters SLCO 2A1 and SLCO2B1 were expressed primarily in endothelial cells, whereas, the transporter SLCO 5A1 was expressed primarily in prostate epithelial cells. On the other hand, expression of SLCO1B1 and 1B3 was very low, or not detected in prostate. SLCO5A1 was found to be involved in non-classical functions [84], however, the critical substrates for SLCO5A1 were not identified. Substrates for SLCO2A1 have not been fully examined, however, prostaglandin is a known substrate and, therefore, SLCO2A1 has been associated with prostaglandin function in endothelial cells [85, 86]. The cell type-specific expression of SLCO2B1, 2A1, and 5A1 indicated distinctive cell type-specific functions of these transporters in human prostate cells. Significantly, since DHEA-S and paclitaxel are SLCO2B1 substrates, endothelial cell-specific expression of SLCO2B1 suggested an important role of prostate endothelial cells in regulation of the entry of DHEA-S and chemotherapeutic agents into prostate tissue. The different expression profiles of ABC pumps, SLC transporters and SLCO transporters in the three cell types of the prostate can provide important insights at the molecular level of the cell type-specific regulation of substrate exchanges between different cell types within prostate tissue, and between prostate tissue and the circulation.

In summary, our data validated that primary prostate tissue xenografts are a reliable model of prostate tissue *in situ* that preserves the critical cellular architectures of the tissue, the transcriptomes of the individual cell types of the tissue, and the intra-cellular signaling that maintains prostate tissue homeostasis or that drives disease progression. The identification of cell type-specific genes sets, including the cell type-specific genes for androgen metabolism, AR signaling associated genes, uptake transporters and efflux pumps provides valuable insights into cell type-specific functions and interactions in benign prostate. Our research is focused on comparison between fresh tissue and tissue xenograft of clinical prostate cancer, and the response to androgen deprivation. In this first report we focused on presentation of the “control model”, benign tissue. Nonetheless, this data will have broad implications understanding normal functions of, and interactions among, different cell types in prostate tissue. Further, it is of interest to investigate the cell type specific response to androgen deprivation, a treatment for both benign and malignant prostate disease.

## Supporting information

S1 Fig Sample inclusion criteria for RNA-Seq data analysis. The x-axis is the total number of processed reads with non-human (mouse) reads removed. The y-axis shows the percentage of processed reads. Samples above the dashed line had at least 1.7 M total processed reads. Four samples below the dashed line were excluded due to failure to meet either/both criteria.
(TIF)

S2 Fig IPA canonical pathway analysis of human prostate epithelial cells versus stromal cells. Pathways are identified as: more active in epithelial cells (Epi), more active in stromal cells (Stroma), not determined due to insufficient knowledge (NA), or not determined due to in-sufficient input (zero). The vertical blue line indicates a *p*-value of 0.05 with -log_10_(*p*-value) to the right of the line indicating smaller *p*-value.
(TIF)

S3 Fig IPA canonical pathway analysis of human prostate endothelial cells versus stromal cells. Pathways are identified as: more active in endothelial cells (Endo), more active in stromal cells (Stroma), not determined due to insufficient knowledge (NA), or not determined due to insufficient input (zero). The vertical blue line indicates a *p*-value of 0.05 with -log_10_(*p*-value) to the right of the line indicating smaller *p*-value.
(TIF)

S4 Fig The fresh tissue xenograft model preserves key features of prostate tissue in situ. Tissue xenografts 1 and 2, each from a different prostate cancer patient, were stained for AR using immunohistochemistry (IHC). Two consecutive serial sections of a clinical prostate cancer tissue specimen were used for a positive control (AR antibody followed with HRP-conjugated secondary antibody) and a negative control (HRP-conjugated secondary antibody only), respectively. Arrow heads indicate glands in xenografts, whereas, arrows indicate columnar luminal epithelial cells in glands in the xenografts. Detailed method for AR IHC is provided in Supplemental Methods.
(TIF)

S1 Table Literature based consensus epithelial markers. (DOCX)

S2 Table Literature based consensus endothelial markers. (DOCX)

S3 Table (DOCX)

S4 Table Cell type-specific genes identified for human prostate endothelial cells (176 genes), epithelial cells (48 genes), and stromal cells (46 genes). (XLSX)

S5 Table IPA canonical pathway analysis of differential expression between epithelial cells and endothelial cells, epithelial cells and stromal cells, and endothelial cells and stromal cells. Results for each comparison are presented in a separate tab. #NUM! values indicate no Z scores could be assigned, which were distinct from a value of zero that meant a Z-score of zero could be assigned but the directions of change could not be determined.
(XLSX)

S6 Table Expression of 173 AR co-regulator genes compared between cell types. Base means are presented in the first tab (“Base mean”). Comparisons between cell types are presented in separate tabs “Epi vs Endo”, “Epi vs Stroma”, and “Endo vs Stroma”.
(XLSX)

S7 Table Expression of 1149 AR-regulated genes compared between cell types. The full gens lists and associated references are presented in the first tab (“AR-regulated genes”). Comparisons between cell types are presented in separate tabs “Endo vs Epi”, “Stroma vs Epi”, and “Endo vs Stroma”.
(XLSX)

S8 Table Selected androgen metabolic enzyme genes and corresponding references. Genes are presented in the order that they appear in Fig 8A and 8B.
(XLSX)

S9 Table Gene names and relevant references for ATP cassette-binding (ABC) efflux pumps (Fig 9A), solute carrier (SLC) transporters (Fig 9B), and solute carrier organic anion (SLCO) transporters (Fig 9C). (XLSX)

S1 Data (DOCX)
