# Supplementary figures and images for: Recapitulation of prostate tissue cell type-specific transcriptomes by an in vivo primary prostate tissue xenograft model
Source: PLoS One. 2020 Jun 25;15(6):e0233899. doi: 10.1371/journal.pone.0233899 (PMC7316257; doi:10.1371/journal.pone.0233899)

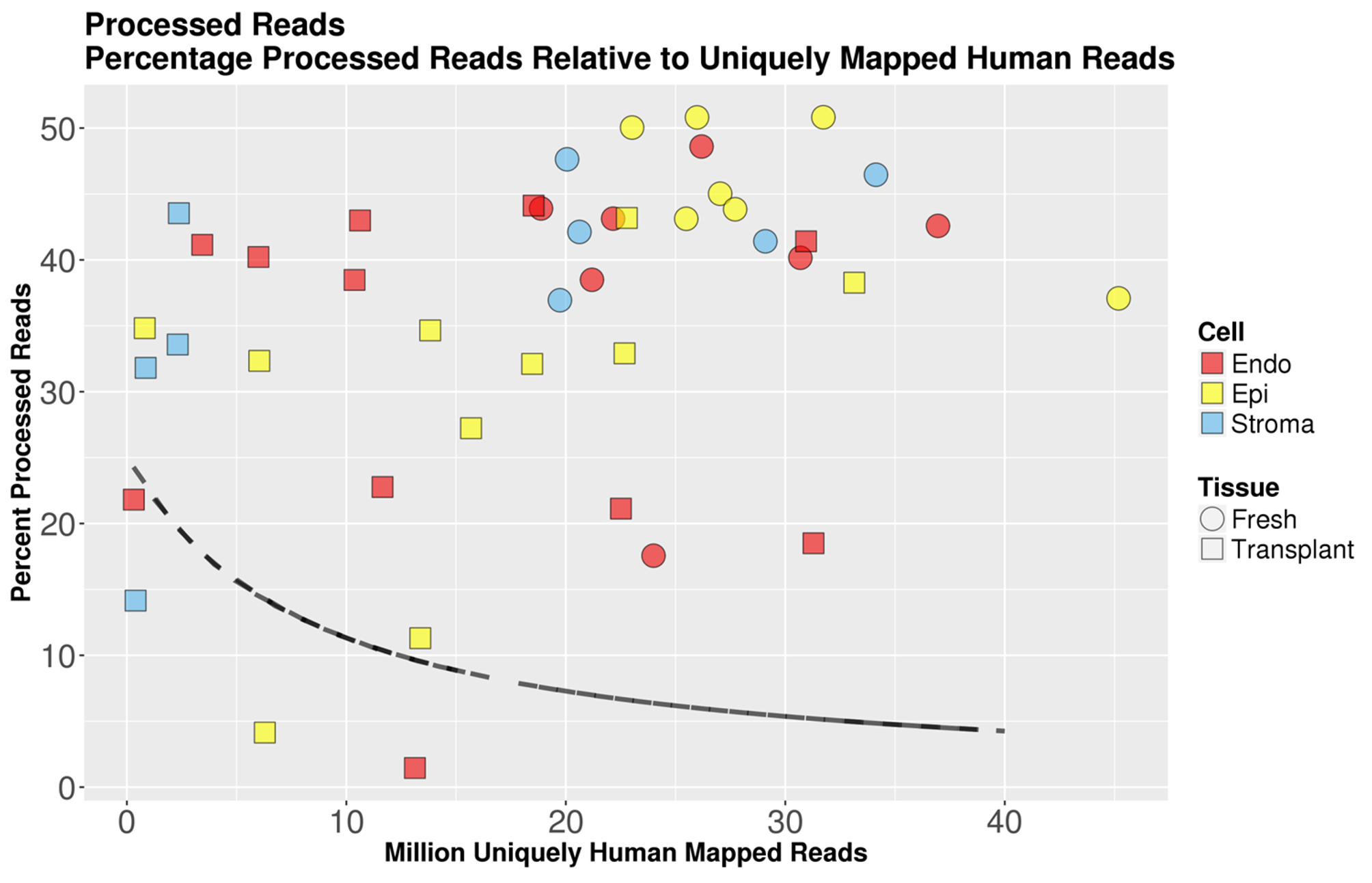

Supplement: S1 Fig — The x-axis is the total number of processed reads with non-human (mouse) reads removed. The y-axis shows the percentage of processed reads. Samples above the dashed line had at least 1.7 M total processed reads. Four samples below the dashed line were excluded due to failure to meet either/both criteria. (TIF) [file pone.0233899.s001.tif]

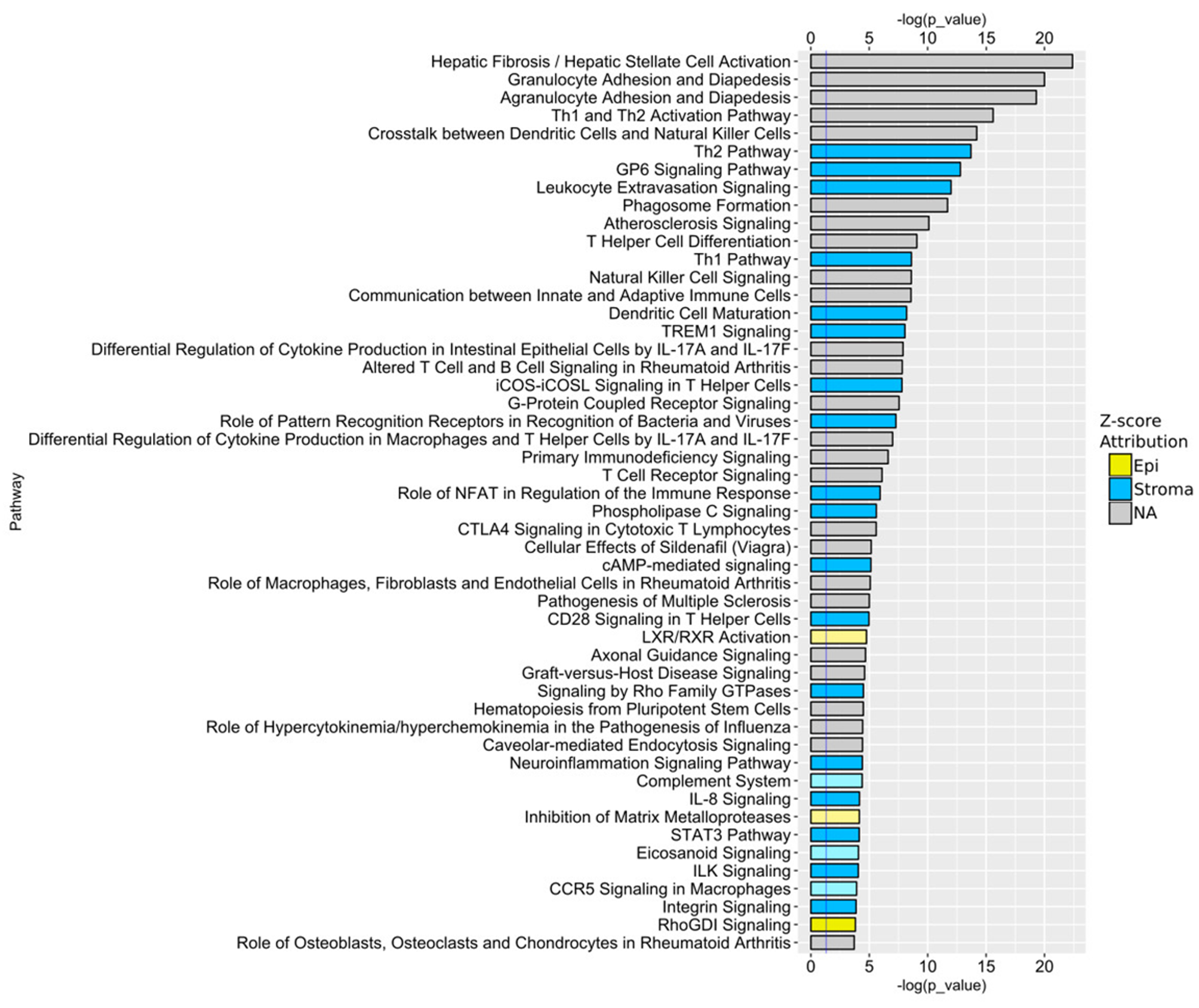

Supplement: S2 Fig — Pathways are identified as: more active in epithelial cells (Epi), more active in stromal cells (Stroma), not determined due to insufficient knowledge (NA), or not determined due to in-sufficient input (zero). The vertical blue line indicates a p-value of 0.05 with -log10(p-value) to the right of the line indicating smaller p-value. (TIF) [file pone.0233899.s002.tif]

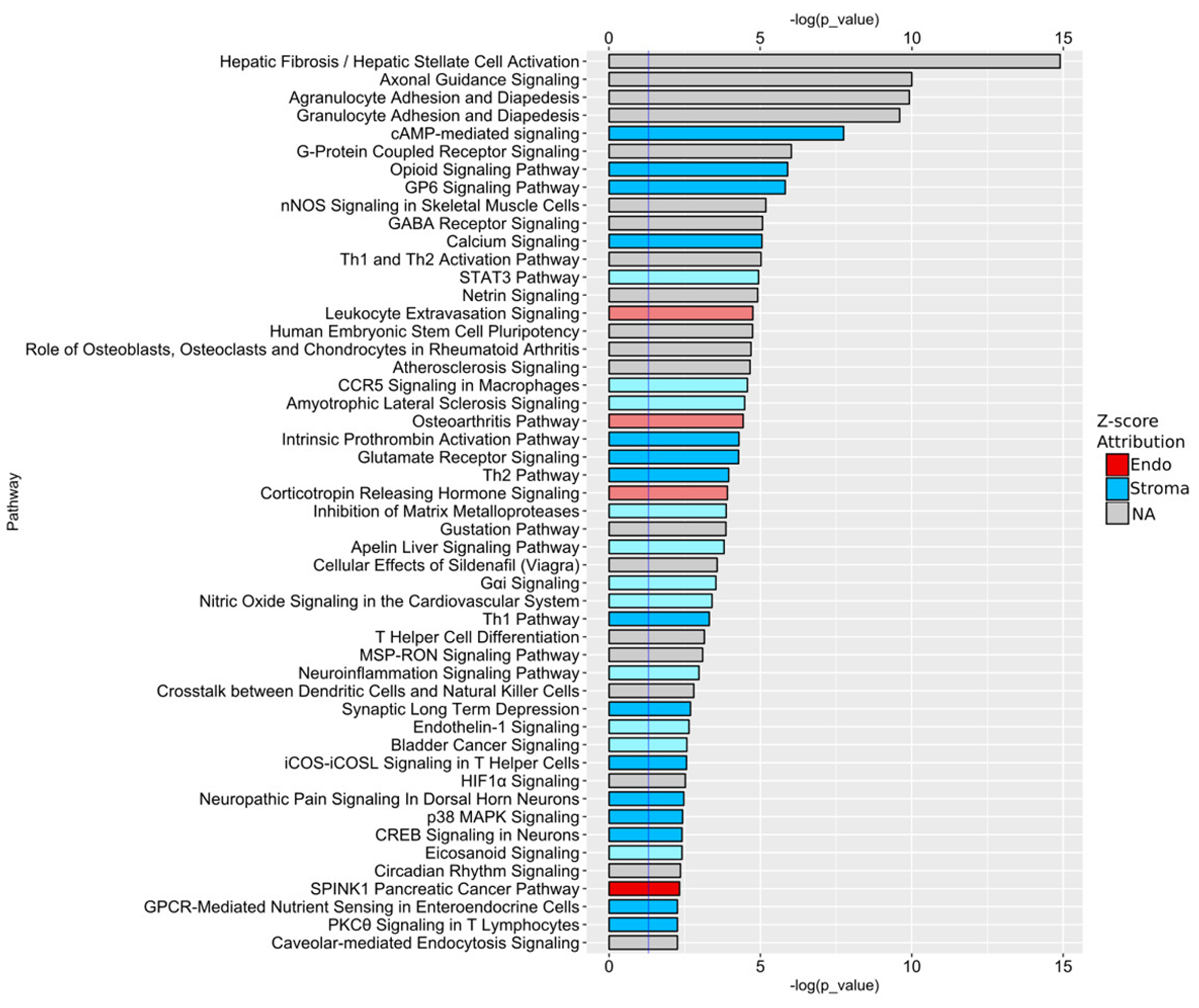

Supplement: S3 Fig — Pathways are identified as: more active in endothelial cells (Endo), more active in stromal cells (Stroma), not determined due to insufficient knowledge (NA), or not determined due to insufficient input (zero). The vertical blue line indicates a p-value of 0.05 with -log10(p-value) to the right of the line indicating smaller p-value. (TIF) [file pone.0233899.s003.tif]

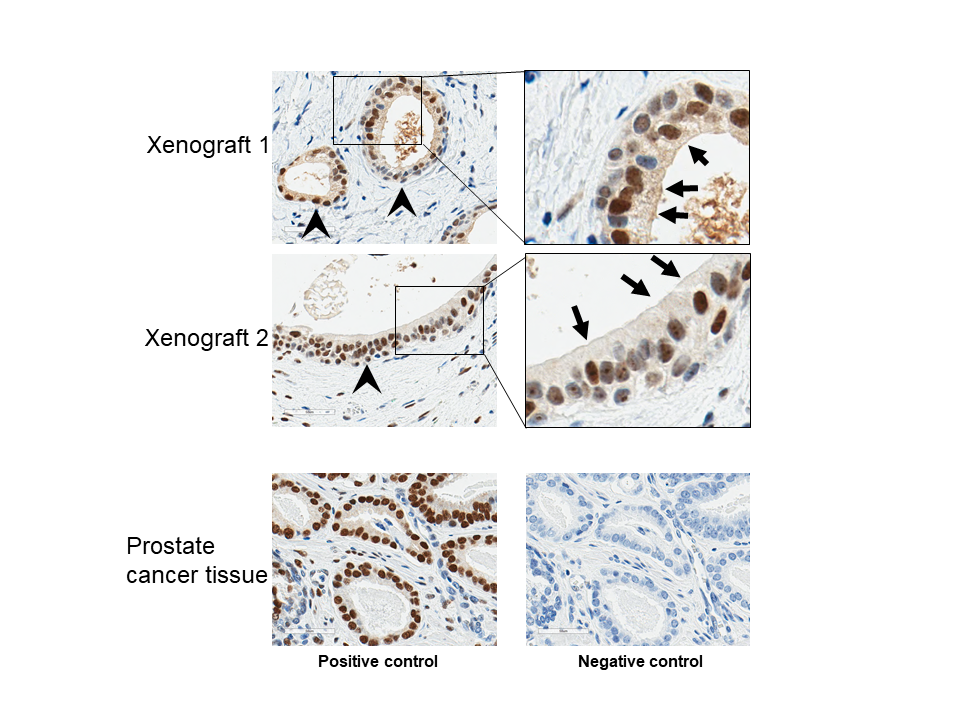

Supplement: S4 Fig — Tissue xenografts 1 and 2, each from a different prostate cancer patient, were stained for AR using immunohistochemistry (IHC). Two consecutive serial sections of a clinical prostate cancer tissue specimen were used for a positive control (AR antibody followed with HRP-conjugated secondary antibody) and a negative control (HRP-conjugated secondary antibody only), respectively. Arrow heads indicate glands in xenografts, whereas, arrows indicate columnar luminal epithelial cells in glands in the xenografts. Detailed method for AR IHC is provided in Supplemental Methods. (TIF) [file pone.0233899.s004.tif]
