# Supplementary material for: Recapitulation of prostate tissue cell type-specific transcriptomes by an in vivo primary prostate tissue xenograft model
Source: PLoS One. 2020 Jun 25;15(6):e0233899. doi: 10.1371/journal.pone.0233899 (PMC7316257; doi:10.1371/journal.pone.0233899)
Supplement: S2 Table — (DOCX) [file pone.0233899.s006.docx]

**Supplementary Table S2.** Literature based consensus endothelial markers.

| Gene | Base Mean | Supplementary Reference |
| --- | --- | --- |
| VWF | 12024.6 | 40 |
| MCAM | 4671.1 | 41 |
| KDR | 2293.5 | 42 |
| CD93 | 6679.2 | 43 |
| FLT1 | 3812.9 | 44 |
| PECAM1 | 2545.7 | 45 |
| CD34 | 1539.6 | 46 |
| CDH5 | 1840.3 | 47 |
| ENG | 758.7 | 48 |
| ESAM | 815.9 | 49 |
| TEK | 548.9 | 50 |
| S1PR1 | 833.9 | 51 |
| LYVE1 | 432.9 | 52 |
| FLT4 | 490.2 | 44 |
| ICAM2 | 310.2 | 53 |
| F3 | 671.5 | 54 |
| VCAM1 | 620.4 | 55 |
| ITGB2 | 368.6 | 56 |
| TNFRSF1B | 351.7 | 57 |
| PTPRC | 1454.3 | 58 |
| IL1R1 | 1273.0 | 59 |
| ICAM1 | 1322.1 | 60 |
| IL13RA1 | 470.2 | 61 |
| TNFRSF1A | 537.2 | 62 |
| BSG | 950.4 | 63 |
| TNFRSF10B | 807.7 | 64 |
| SELE | 3439.1 | 65 |
| SELP | 437.1 | 66 |
| ACKR2 | 15.3 | 67 |
| CD160 | 5.2 | 68 |
| STAB2 | 5.9 | 69 |
| CLEC4M | 0.6 | 70 |
| EGFL8 | 0.9 | 71 |
| CD36 | 224.0 | 76 |
| FABP5 | 142.1 | 77 |
| PROCR | 107.5 | 78 |
| TNFRSF10A | 114.7 | 79 |
| SELPLG | 82.5 | 66 |
| PDPN | 67.0 | 72 |
| EPOR | 82.4 | 73 |
| CLP1 | 60.2 | 74 |
| S1PR2 | 58.6 | 75 |

**Supplementary references for tables S1 and S2**

1 Wagner, N., et al., A splice variant of the Wilms' tumour suppressor Wt1 is required for normal development of the olfactory system. Development, 2005. 132(6): p. 1327-1336.

2 Kasper, M., et al., Monoclonal antibodies to surfactant protein D: evaluation of immunoreactivity in normal rat lung and in a radiation-induced fibrosis model. Experimental lung research, 1995. 21(4): p. 577-588.

3 Gregory, T., et al., Surfactant chemical composition and biophysical activity in acute respiratory distress syndrome. The Journal of clinical investigation, 1991. 88(6): p. 1976-1981.

4 Wild, G.-A. and D. Mischke, Variation and frequency of cytokeratin polypeptide patterns in human squamous non-keratinizing epithelium. Experimental cell research, 1986. 162(1): p. 114-126.

5 Moll, R., et al., The catalog of human cytokeratins: patterns of expression in normal epithelia, tumors and cultured cells. Cell, 1982. 31(1): p. 11-24.

6 Szabat, E., et al., Production and characterization of a monoclonal antibody against human calcitonin gene-related peptide (CGRP) and its immunohistochemical application to salivary glands. The Histochemical Journal, 1994. 26(4): p. 317-326.

7 Sun, T.-T. and H. Green, Immunofluorescent staining of keratin fibers in cultured cells. Cell, 1978. 14(3): p. 469-476.

8 Bellini, A., et al., Interleukin (IL)-4, IL-13, and IL-17A differentially affect the profibrotic and proinflammatory functions of fibrocytes from asthmatic patients. Mucosal immunology, 2012. 5(2): p. 140.

9 Allory, Y., et al., The L1 cell adhesion molecule is a potential biomarker of human distal nephron injury in acute tubular necrosis. Kidney international, 2008. 73(6): p. 751-758.

10 Schnickmann, S., et al., AP-1-controlled hepatocyte growth factor activation promotes keratinocyte migration via CEACAM1 and urokinase plasminogen activator/urokinase plasminogen receptor. Journal of Investigative Dermatology, 2009. 129(5): p. 1140-1148.

11 Waseem, A., et al., Keratin 15 expression in stratified epithelia: downregulation in activated keratinocytes. Journal of investigative dermatology, 1999. 112(3): p. 362-369.

12 Calon, A., et al., Stromal gene expression defines poor-prognosis subtypes in colorectal cancer. Nature genetics, 2015. 47(4): p. 320.

13 Noyce, R.S., et al., Tumor cell marker PVRL4 (nectin 4) is an epithelial cell receptor for measles virus. PLoS pathogens, 2011. 7(8): p. e1002240.

14 Gebauer, F., et al., Establishment and Characterization of a Pair of Patient-derived Human Non-small Cell Lung Cancer Cell Lines from a Primary Tumor and Corresponding Lymph Node Metastasis. Anticancer research, 2016. 36(4): p. 1507-1518.

15 Pavlova, N.N., et al., A role for PVRL4-driven cell–cell interactions in tumorigenesis. Elife, 2013. 2: p. e00358.

16 Vojtĕsek, B., et al., A panel of monoclonal antibodies to keratin no. 7: characterization and value in tumor diagnosis. Neoplasma, 1990. 37(3): p. 333-342.

17 Nuber, U., et al., Patterns of desmocollin synthesis in human epithelia: immunolocalization of desmocollins 1 and 3 in special epithelia and in cultured cells. European journal of cell biology, 1996. 71(1): p. 1-13.

18 Ciocca, D. and E. Luque, Immunological evidence for the identity between the hsp27 estrogen-regulated heat shock protein and the p29 estrogen receptor-associated protein in breast and endometrial cancer. Breast cancer research and treatment, 1991. 20(1): p. 33-42.

19 Harris, B., I. Pereira, and E. Parkin, Targeting ADAM10 to lipid rafts in neuroblastoma SH-SY5Y cells impairs amyloidogenic processing of the amyloid precursor protein. Brain research, 2009. 1296: p. 203-215.

20 Johnson, J.B., K. Grant, and G.D. Parks, The paramyxoviruses simian virus 5 and mumps virus recruit host cell CD46 to evade complement-mediated neutralization. Journal of virology, 2009. 83(15): p. 7602-7611.

21 Makino, A., et al., Junctional adhesion molecule 1 is a functional receptor for feline calicivirus. Journal of virology, 2006. 80(9): p. 4482-4490.

22 Stanton, L.-A. and F. Beier, Inhibition of p38 MAPK signaling in chondrocyte cultures results in enhanced osteogenic differentiation of perichondral cells. Experimental cell research, 2007. 313(1): p. 146-155.

23 Bartek, J., et al., Patterns of expression of keratin 19 as detected with monoclonal antibodies in human breast tissues and tumours. International journal of cancer, 1985. 36(3): p. 299-306.

24 Guelstein, V., et al., Monoclonal antibody mapping of keratins 8 and 17 and of vimentin in normal human mammary gland, benign tumors, dysplasias and breast cancer. International journal of cancer, 1988. 42(2): p. 147-153.

25 Moll, I. and R. Moll, Comparative cytokeratin analysis of sweat gland ducts and eccrine poromas. Archives of dermatological research, 1991. 283(5): p. 300-309.

26 Hitomi, J., et al., Development of a novel monoclonal antibody recognizing basal cells of human squamous epithelia. Archives of histology and cytology, 2002. 65(2): p. 201-208.

27 Jubeck, B., et al., Type II collagen levels correlate with mineralization by articular cartilage vesicles. Arthritis & Rheumatism, 2009. 60(9): p. 2741-2746.

28 Kutleša, S., et al., E-cadherin-mediated interactions of thymic epithelial cells with CD103+ thymocytes lead to enhanced thymocyte cell proliferation. Journal of Cell Science, 2002. 115(23): p. 4505-4515.

29 Petroff, M.G., et al., The immunomodulatory proteins B7-DC, B7-H2, and B7-H3 are differentially expressed across gestation in the human placenta. The American journal of pathology, 2005. 167(2): p. 465-473.

30 Heijink, I.H., et al., Der p, IL-4, and TGF-β cooperatively induce EGFR-dependent TARC expression in airway epithelium. American journal of respiratory cell and molecular biology, 2007. 36(3): p. 351-359.

31 Zen, K., et al., JAM-C is a component of desmosomes and a ligand for CD11b/CD18-mediated neutrophil transepithelial migration. Molecular biology of the cell, 2004. 15(8): p. 3926-3937.

32 Stratman, A.N., et al., Pericyte recruitment during vasculogenic tube assembly stimulates endothelial basement membrane matrix formation. Blood, 2009. 114(24): p. 5091-5101.

33 Gaggianesi, M., et al., IL4 primes the dynamics of breast Cancer progression via DUSP4 inhibition. Cancer research, 2017.

34 Thobe, M.N., et al., The Ron receptor tyrosine kinase positively regulates angiogenic chemokine production in prostate cancer cells. Oncogene, 2010. 29(2): p. 214.

35 Ambrosini, G., C. Adida, and D.C. Altieri, A novel anti-apoptosis gene, survivin, expressed in cancer and lymphoma. Nature medicine, 1997. 3(8): p. 917-921.

36 Broekaert, D., et al., An investigation of cytokeratin expression in skin epithelial cysts and some uncommon types of cystic tumours using chain-specific antibodies. Archives of dermatological research, 1990. 282(6): p. 383-391.

37 Nagasawa, K., et al., Possible involvement of gap junctions in the barrier function of tight junctions of brain and lung endothelial cells. Journal of cellular physiology, 2006. 208(1): p. 123-132.

38 Uchiyama, A., et al., MFG-E8 regulates angiogenesis in cutaneous wound healing. The American journal of pathology, 2014. 184(7): p. 1981-1990.

39 Moiseeva, E.P., et al., CADM1 is a key receptor mediating human mast cell adhesion to human lung fibroblasts and airway smooth muscle cells. PLoS One, 2013. 8(4): p. e61579.

40 Al Asoom, L.I., Coronary angiogenic effect of long-term administration of Nigella sativa. BMC complementary and alternative medicine, 2017. 17(1): p. 308.

41 Shih, I.M., The role of CD146 (Mel‐CAM) in biology and pathology. The Journal of pathology, 1999. 189(1): p. 4-11.

42 Dellinger, M.T. and R.A. Brekken, Phosphorylation of Akt and ERK1/2 is required for VEGF-A/VEGFR2-induced proliferation and migration of lymphatic endothelium. PloS one, 2011. 6(12): p. e28947.

43 Mälarstig, A., et al., Plasma CD93 concentration is a potential novel biomarker for coronary artery disease. Journal of internal medicine, 2011. 270(3): p. 229-236.

44 Valtola, R., et al., VEGFR-3 and its ligand VEGF-C are associated with angiogenesis in breast cancer. The American journal of pathology, 1999. 154(5): p. 1381-1390.

45 Cabañas, C., et al., Characterization of a novel myeloid antigen regulated during differentiation of monocytic cells. European journal of immunology, 1989. 19(8): p. 1373-1378.

46 Fina, L., et al., Expression of the CD34 gene in vascular endothelial cells. Blood, 1990. 75(12): p. 2417-2426.

47 Esser, S., et al., Vascular endothelial growth factor induces VE-cadherin tyrosine phosphorylation in endothelial cells. Journal of cell science, 1998. 111(13): p. 1853-1865.

48 Batlle, R., et al., Snail1 controls TGF-β responsiveness and differentiation of mesenchymal stem cells. Oncogene, 2013. 32(28): p. 3381.

49 Roesli, C., et al., Identification of the surface-accessible, lineage-specific vascular proteome by two-dimensional peptide mapping. The FASEB Journal, 2008. 22(6): p. 1933-1944.

50 Marron, M.B., et al., Evidence for heterotypic interaction between the receptor tyrosine kinases TIE-1 and TIE-2. Journal of Biological Chemistry, 2000. 275(50): p. 39741-39746.

51 Lorquet, S., et al., Soluble forms of VEGF receptor-1 and-2 promote vascular maturation via mural cell recruitment. The FASEB Journal, 2010. 24(10): p. 3782-3795.

52 Shawber, C.J., et al., Notch alters VEGF responsiveness in human and murine endothelial cells by direct regulation of VEGFR-3 expression. The Journal of clinical investigation, 2007. 117(11): p. 3369-3382.

53 Silverman, M.D., et al., The role of vascular cell adhesion molecule 1/very late activation antigen 4 in endothelial progenitor cell recruitment to rheumatoid arthritis synovium. Arthritis & Rheumatism, 2007. 56(6): p. 1817-1826.

54 Conroy, A.L., et al., Performance characteristics of combinations of host biomarkers to identify women with occult placental malaria: a case-control study from Malawi. PloS one, 2011. 6(12): p. e28540.

55 Elliott, D.A., et al., NKX2-5 eGFP/w hESCs for isolation of human cardiac progenitors and cardiomyocytes. Nature methods, 2011. 8(12): p. 1037.

56 Van Grevenstein, W., et al., Inflammatory cytokines stimulate the adhesion of colon carcinoma cells to mesothelial monolayers. Digestive diseases and sciences, 2007. 52(10): p. 2775-2783.

57 Jyonouchi, H., S. Sun, and N. Itokazu, Innate immunity associated with inflammatory responses and cytokine production against common dietary proteins in patients with autism spectrum disorder. Neuropsychobiology, 2002. 46(2): p. 76-84.

58 Horejsi, V., et al., Monoclonal antibodies against human leucocyte antigens. II. Antibodies against CD45 (T200), CD3 (T3), CD43, CD10 (CALLA), transferrin receptor (T9), a novel broadly expressed 18-kDa antigen (MEM-43) and a novel antigen of restricted expression (MEM-74). Folia biologica, 1988. 34(1): p. 23-34.

59 Herrmann-Lavoie, C., C. Rao, and A. Akoum, Chorionic gonadotropin down-regulates the expression of the decoy inhibitory interleukin 1 receptor type II in human endometrial epithelial cells. Endocrinology, 2007. 148(11): p. 5377-5384.

60 Malara, N., et al., Soluble CD54 induces human endothelial cells ex vivo expansion useful for cardiovascular regeneration and tissue engineering application. IJC Heart & Vasculature, 2015. 6: p. 48-53.

61 Zhou, Y., et al., Vascular endothelial growth factor ligands and receptors that regulate human cytotrophoblast survival are dysregulated in severe preeclampsia and hemolysis, elevated liver enzymes, and low platelets syndrome. The American journal of pathology, 2002. 160(4): p. 1405-1423.

62 Mizrahi, K., et al., Negative selection by apoptosis enriches progenitors in naïve and expanded human umbilical cord blood grafts. Bone marrow transplantation, 2014. 49(7): p. 942.

63 Haug, C., et al., Oxidized low-density lipoproteins stimulate extracellular matrix metalloproteinase Inducer (EMMPRIN) release by coronary smooth muscle cells. Arteriosclerosis, thrombosis, and vascular biology, 2004. 24(10): p. 1823-1829.

64 Bretz, J., et al., Inflammatory cytokine regulation of TRAIL-mediated apoptosis in thyroid epithelial cells. Cell death and differentiation, 2002. 9(3): p. 274.

65 Rémy-Zolghadri, M., et al., Endothelium properties of a tissue-engineered blood vessel for small-diameter vascular reconstruction. Journal of vascular surgery, 2004. 39(3): p. 613-620.

66 Lalor, P.F., et al., Vascular adhesion protein-1 mediates adhesion and transmigration of lymphocytes on human hepatic endothelial cells. The Journal of Immunology, 2002. 169(2): p. 983-992.

67 de la Torre, Y.M., et al., Protection against inflammation-and autoantibody-caused fetal loss by the chemokine decoy receptor D6. Proceedings of the National Academy of Sciences, 2007. 104(7): p. 2319-2324.

68 El-Far, M., et al., CD160 isoforms and regulation of CD4 and CD8 T-cell responses. Journal of translational medicine, 2014. 12(1): p. 217.

69 WAUTIER, M.P., et al., Red blood cell phosphatidylserine exposure is responsible for increased erythrocyte adhesion to endothelium in central retinal vein occlusion. Journal of thrombosis and haemostasis, 2011. 9(5): p. 1049-1055.

70 Falkowska, E., et al., L-SIGN (CD209L) isoforms differently mediate trans-infection of hepatoma cells by hepatitis C virus pseudoparticles. Journal of general virology, 2006. 87(9): p. 2571-2576.

71 Lelievre, E., et al., VE‐statin/egfl7 regulates vascular elastogenesis by interacting with lysyl oxidases. The EMBO journal, 2008. 27(12): p. 1658-1670.

72 Yuan, J.X., et al., Quantitative analysis of the cellular microenvironment of glioblastoma to develop predictive statistical models of overall survival. Journal of Neuropathology & Experimental Neurology, 2016: p. nlw090.

73 Pontikoglou, C., et al., Evidence for downregulation of erythropoietin receptor in bone marrow erythroid cells of patients with chronic idiopathic neutropenia. Experimental hematology, 2006. 34(10): p. 1312-1322.

74 Keuschnigg, J., et al., Plasticity of blood-and lymphatic endothelial cells and marker identification. PloS one, 2013. 8(9): p. e74293.

75 Capitani, N., et al., S1P1 expression is controlled by the pro-oxidant activity of p66Shc and is impaired in B-CLL patients with unfavorable prognosis. Blood, 2012: p. blood-2012-04-425959.

76 Mahadevan, V.S., et al., Internal mammary artery smooth muscle cells resist migration and possess high antioxidant capacity. Cardiovascular research, 2006. 72(1): p. 60-68.

77 Kannan-Thulasiraman, P., et al., Fatty acid binding protein 5 and PPARβ/δ are critical mediators of EGFR-induced carcinoma cell growth. Journal of Biological Chemistry, 2010: p. jbc. M109. 099770.

78 Xue, M., D. Campbell, and C.J. Jackson, Protein C is an autocrine growth factor for human skin keratinocytes. Journal of Biological Chemistry, 2007. 282(18): p. 13610-13616.

79 Singh, T.R., S. Shankar, and R.K. Srivastava, HDAC inhibitors enhance the apoptosis-inducing potential of TRAIL in breast carcinoma. Oncogene, 2005. 24(29): p. 4609.
