# Supplementary material for: Recapitulation of prostate tissue cell type-specific transcriptomes by an in vivo primary prostate tissue xenograft model
Source: PLoS One. 2020 Jun 25;15(6):e0233899. doi: 10.1371/journal.pone.0233899 (PMC7316257; doi:10.1371/journal.pone.0233899)
Supplement: S3 Table — (DOCX) [file pone.0233899.s007.docx]

| **Sample ID** | **Sources of cell-type specific enriched cell fractions** | | | | | |
| --- | --- | --- | --- | --- | --- | --- |
|  | **Fresh tissue** | | | **Primary tissue xenograft** | | |
|  | **Epithelial cells** | **Endothelial cells** | **Stromal**  **cells** | **Epithelial cells** | **Endothelial cells** | **Stromal**  **cells** |
| **HP1084** | **+** | **+** |  |  |  |  |
| **HP1096** | **+** | **+** |  |  |  |  |
| **HP1140** | **+** | **+** | **+** |  |  |  |
| **HP1145** | **+** | **+** | **+** |  |  |  |
| **HP1146** | **+** | **+** | **+** |  |  |  |
| **HP1147** | **+** | **+** | **+** |  |  |  |
| **HP1155** | **+** | **+** | **+** |  |  |  |
| **HP1085** |  |  |  | **+** | **+** |  |
| **HP1087** |  |  |  | **+** | **+** |  |
| **HP1091** |  |  |  | **+** | **+** |  |
| **HP1095** |  |  |  | **+^a^** | **+^a^** |  |
| **HP1097** |  |  |  | **+** | **+** |  |
| **HP1098** |  |  |  | **+** | **+** |  |
| **HP1099** |  |  |  | **+** | **+** |  |
| **HP1104** |  |  |  | **+** | **+** | **+** |
| **HP1109** |  |  |  | **+** | **+** | **+** |
| **HP1125** |  |  |  |  | **+^a^** | **+^a^** |
| **HP1131** |  |  |  | **+** | **+** | **+** |
| **Total number of samples** | **7** | **7** | **5** | **10** | **11** | **4** |
| **Total number of further analyzed samples** | **7** | **7** | **5** | **9** | **9** | **3** |
| **^a^:excluded after quality control for mouse gene readings** | | | | | | |
