# Supplementary material for: Recapitulation of prostate tissue cell type-specific transcriptomes by an in vivo primary prostate tissue xenograft model
Source: PLoS One. 2020 Jun 25;15(6):e0233899. doi: 10.1371/journal.pone.0233899 (PMC7316257; doi:10.1371/journal.pone.0233899)
Supplement: S1 Data — (DOCX) [file pone.0233899.s014.docx]

Supplementary Methods:

**Immunohistochemistry (IHC) of AR.** Formalin-fixed paraffin embedded (FFPE) tissue blocks were cut into 4 µm sections, placed on charged slides, and dried at 60°C for one hour. Slides were cooled to room temperature, and deparaffinized with Clearify (American Mastertech, Lodi, CA) and rinsed in water in a Dako Omnis autostainer (Agilent Technologies). Epitopes were retrieved with treatment with Dako Flex TRS Low (Agilent Technologies) for 30 minutes. Slides were incubated with 1:200 diluted Abcam ab74272 AR antibody (Abcam, Cambridge, MA) for 30 minutes, followed by Dako GV823 HRP-conjugated secondary antibody (Agilent Technologies) for 20 mins. DAB (Diaminobenzidine) (Agilent Technologies) was applied for 5 minutes for visualization. Slides were counterstained with Hematoxylin for 8 minutes, rinsed with water, dehydrated, cleared and mounted with coverslips. Images of the stained sections were acquired using an Aperio ScanScope XT (Leica Biosystems, Buffalo Grove, IL). Images were processed using Aperio eSlide Manager.
